# Assessment tools for unrecognized myocardial infarction: a cross-sectional analysis of the REasons for geographic and racial differences in stroke population

**DOI:** 10.1186/1471-2261-13-23

**Published:** 2013-03-26

**Authors:** Emily B Levitan, Monika M Safford, Meredith L Kilgore, Elsayed Z Soliman, Stephen P Glasser, Suzanne E Judd, Paul Muntner

**Affiliations:** 1 Department of Epidemiology, University of Alabama at Birmingham, Birmingham, AL, USA; 2 Division of Preventive Medicine, University of Alabama at Birmingham, Birmingham, AL, USA; 3 Department of Healthcare Organization and Policy, University of Alabama at Birmingham, Birmingham, AL, USA; 4 Epidemiological Cardiology Research Center, Wake Forest University School of Medicine, Winston Salem, NC, USA; 5 Department of Biostatistics, University of Alabama at Birmingham, Birmingham, AL, USA

**Keywords:** Myocardial infarction, Screening, Electrocardiography

## Abstract

**Background:**

Routine electrocardiograms (ECGs) are not recommended for asymptomatic patients because the potential harms are thought to outweigh any benefits. Assessment tools to identify high risk individuals may improve the harm versus benefit profile of screening ECGs. In particular, people with unrecognized myocardial infarction (UMI) have elevated risk for cardiovascular events and death.

**Methods:**

Using logistic regression, we developed a basic assessment tool among 16,653 participants in the REasons for Geographic and Racial Differences in Stroke (REGARDS) study using demographics, self-reported medical history, blood pressure, and body mass index and an expanded assessment tool using information on 51 potential variables. UMI was defined as electrocardiogram evidence of myocardial infarction without a self-reported history (n = 740).

**Results:**

The basic assessment tool had a c-statistic of 0.638 (95% confidence interval 0.617 - 0.659) and included age, race, smoking status, body mass index, systolic blood pressure, and self-reported history of transient ischemic attack, deep vein thrombosis, falls, diabetes, and hypertension. A predicted probability of UMI > 3% provided a sensitivity of 80% and a specificity of 30%. The expanded assessment tool had a c-statistic of 0.654 (95% confidence interval 0.634-0.674). Because of the poor performance of these assessment tools, external validation was not pursued.

**Conclusions:**

Despite examining a large number of potential correlates of UMI, the assessment tools did not provide a high level of discrimination. These data suggest defining groups with high prevalence of UMI for targeted screening will be difficult.

## Background

Electrocardiogram (ECG) screening may identify previously unrecognized, treatable cardiac problems, such as unrecognized myocardial infarction (UMI), thereby improving health, but could also expose patients to side-effects of treatment and lead to further testing with associated costs and risks. Avoiding screening ECGs for low risk individuals has been identified as one of the “top 5 activities” for improving the quality of care in family and internal medicine [1]. Approximately 1-6% of the population without a known history of myocardial infarction has evidence of MI on ECG examination [2]. If UMIs are detected, patients can be treated with medications that have demonstrated benefits for secondary prevention such as aspirin, beta-blockers, renin-angiotensin system blocking agents, and statins [3]. If a subpopulation with high prevalence of UMI could be identified, ECG screening could be targeted efficiently. We therefore sought to develop assessment tools for UMI which could be used to identify populations where ECG screening may be warranted.

## Methods

### Study population

The assessment tools were derived using baseline data from the REasons for Geographic And Racial Differences in Stroke (REGARDS) study population. REGARDS is a nation-wide prospective cohort study of 30,239 men and women aged 45 and older. Details of the study design and recruitment have been previously published [4]. REGARDS participants were recruited between January 2003 and October 2007. Participants were identified using commercially available lists with the goal of equal representation of male and female and African-American and white participants. Geographically, participants were recruited to obtain a population with 20% residing in coastal North Carolina, South Carolina, and Georgia (Stroke Buckle), 30% from the remaining areas of North Carolina, South Carolina, and Georgia plus Tennessee, Mississippi, Alabama, Louisiana, and Arkansas (Stroke Belt), and 50% from the other 40 contiguous US states and the District of Columbia [4].

Potential participants were contacted by telephone. After verbal consent was obtained, data including demographic and socioeconomic factors, medical history, psychosocial factors, lifestyle factors, and cognitive function were collected using computer-assisted telephone interviews (see Additional file 1 for the data collection forms). Following the interview, a technician conducted an in-home physical exam, collected blood and urine, and measured anthropometrics. Written informed consent was obtained during the home visit. A food-frequency questionnaire and a family history questionnaire were left with participants to be completed and returned by mail. This research was conducted in accordance with the Declaration of Helsinki. The REGARDS study was approved by the Institutional Review Boards of the University of Alabama at Birmingham, the University of Vermont, Wake Forest University, and the University of Cincinnati.

A modified 7-lead ECG was used until April 2004, and after this point a standard 12-lead ECG was obtained [4]. Because 7-lead ECGs miss anterior and some lateral MIs, we excluded 9,043 participants without a 12-lead ECG. We additionally excluded participants who reported a history of myocardial infarction or revascularization procedures (n = 2,919). Finally, we excluded individuals who had missing data on potential correlates of UMI (n = 1,624) in order to conduct a complete-case analysis. After these exclusions, there were 16,653 participants with data available for development of the assessment tools.

### Measurement of covariates

The REGARDS data collection instruments contained several standard, previously validated questionnaires used in health research, and additional items selected from questionnaires used in prior studies such as the Behavioral Risk Factor Surveillance System, the Atherosclerosis Risk In Communities Study, the Multi-Ethnic Study of Atherosclerosis, and the Greater Cincinnati/Northern Kentucky Stroke Study [4]. Pilot testing was conducted to ascertain the feasibility of the study processes in the target population [4]. Participants self-reported information about demographics and health behaviors. Urbanization was determined by the census tract in which the participant resided and was categorized as < 25% urban, 25%-75% urban, and >75% urban. Educational attainment was categorized as less than high school, high school graduate, some college, or college graduate or above. Income was categorized < $20,000, $20,000-$34,999, $35,000-$74,999, ≥ $75,000 per year, or refused. Participants answered a single question about current health insurance (yes or no). Participants self-reported regular aspirin use (yes or no) and time spent watching television or videos (none, 1–6 hours/week, 1 hour/day, 2 hours/day, 3 hours/day, or 4 or more hours/day). They additionally reported information about alcohol consumption, cigarette smoking, and exercise. Alcohol consumption was classified as none, moderate (0–7 drinks per week for women and 0–14 drinks per week for men), or heavy (>7 drinks per week for women and >14 drinks per week for men). Cigarette smoking was categorized as current, past, and never. Exercise was categorized as none, 1–3 times per week, or 4 or more times per week. Medication adherence was assessed using the 4-item Morisky scale [5]. Each item in the Morisky scale was considered separately.

Clinical measurements were obtained during the in-home study visit by trained technicians. Body mass index was calculated using measured height and weight. Two blood pressure measurements were taken, and the values averaged. The technicians obtained blood and urine specimens which were processed and shipped to a central laboratory. HDL cholesterol, LDL cholesterol, triglycerides, C-reactive protein, white blood cell count, and serum creatinine were measured in the blood samples. Albumin and creatinine were measured in the urine. Estimated glomerular filtration rate (eGFR) was calculated using the Chronic Kidney Disease Epidemiology Collaboration (CKD-EPI) equation [6]. Because of the nonlinear relationship of eGFR with adverse health effects, we categorized it as ≥90 mL/min/1.73 m^2^, 60–89 mL/min/1.73 m^2^, 45–59 mL/min/1.73 m^2^, 30–44 mL/min/1.73 m^2^, and <30 mL/min/1.73 m^2^. Similarly, the urinary albumin to creatinine ratio was categorized as < 30 mg/g, 30–300 mg/g, and >300 mg/g. Heart rate was measured by ECG. The 6-item screener for cognitive function was administered in the telephone interview [7]. A score of 4 or less was considered cognitive impairment.

Participants reported their history of stroke, transient ischemic attack, deep vein thrombosis, peripheral vascular disease (amputation or surgery), dialysis for kidney failure, falls, diabetes, hypertension, and dyslipidemia. They also reported whether they were currently taking antihypertensive medications. Family history of MI was defined as report of MI in the mother or father of the participant regardless of age at the time of the event. We created variables for unrecognized diabetes, hypertension, and hyperlidipidemia. A participant was considered to have unrecognized diabetes if they reported no prior diagnosis, but had fasting serum glucose ≥126 mg/dL or nonfasting serum glucose ≥200 mg/dL. Similarly, participants were considered to have unrecognized hypertension if they reported no prior diagnosis, but had systolic/diastolic blood pressure ≥140/90 mmHg. Unrecognized dyslipidemia was defined as no self-reported history and total cholesterol ≥240 mg/dL, LDL cholesterol ≥ 160 mg/dL, or HDL cholesterol ≤ 40 mg/dL.

Self-reported health scales included overall health status (as excellent, very good, good, fair, or poor), the 4-item Cohen’s Perceived Stress Scale [8], the 4-item Center for Epidemiologic Studies Depression Scale (CESD-4) [9], and the Medical Outcomes Study Short Form-12 (SF-12) from which physical and mental component scores were calculated [10]. Participants self-reported stroke symptoms, using the Questionnaire for Verifying Stroke-Free Status [11], and whether they needed two or more pillows to sleep at night or woke during the night because of breathlessness.

### Electrocardiogram reading and coding

ECGs performed during the in-home visit were read and coded by a central reading center at Wake Forest University by research staff blinded to other study data. Research staff received initial training and certification followed by quarterly quality control examinations with retraining and recertification when necessary. ECGs were read by a certified coder, followed by a review by a senior coder, and a final review by the center PI (EZS) which focused on major abnormalities. Disagreements were resolved through discussion between the coder, senior coder, and PI. When necessary, a senior coder who had not previously read the ECG in question was asked to resolve the debate. Participants were considered to have UMI if they reported no history of MI, but had evidence of MI on ECG. This included major Q/QS wave abnormalities by the Minnesota code (MC) scheme (MC 1-1-X through 1-2-X) or smaller Q/QS wave abnormalities (MC 1-3-X) with major ST-segment or T-wave abnormalities (MC 4–1, 4–2, 5–1 or 5–2) [12].

### Statistical analysis

We computed means and standard deviations or percentages for each of the potential correlates for participants with and without UMI. We additionally calculated each participant’s Framingham risk score for coronary heart disease [13]. The groups with and without UMI were compared using linear regression for continuous variables and χ^2^ tests for categorical variables. Using logistic regression, we calculated the crude odds ratio and the c-statistic for each potential correlate. We used a logistic regression approach to create the basic assessment tool. Basic demographics (age, sex, and race), smoking and alcohol consumption, self-report of medical history, family history of MI, and basic clinical measurements (body mass index and blood pressure) available during routine office visits were entered into a logistic regression model. For continuous variables, the linear term and the square of the centered value were entered into the model. We then used a backward selection procedure to create a more parsimonious model, requiring a p < 0.20 for a variable to remain. Variables which were removed from the model through backward selection were reconsidered, and if they improved the c-statistic by ≥ 0.005, they were retained. The model was used to calculate the predicted probability of UMI for each participant, and a receiver operating characteristic curve was constructed. We examined the performance of the UMI assessment tools in subgroups defined by sex, race, and age (< 65 years or ≥ 65 years). The procedure was repeated for the expanded set of potential correlates described above including demographics, health behaviors, clinical measurements, medical history, participant reported health scales, and participant reported symptoms (expanded assessment tool). The ability of the Framingham risk score for coronary heart disease to discriminate between those with and without UMI was evaluated using the c-statistic and receiver operating characteristic curve. Analysis was conducted using SAS version 9.2 (Cary, NC), and p values < 0.05 were considered statistically significant.

## Results

### Population characteristics

In the REGARDS study population without a reported history of MI, 740 participants had UMI (4.4%). Characteristics of the REGARDS study population considered for the basic assessment tool are presented in Table 1 by UMI status. The factors in the expanded assessment tool are presented in Additional file 2: Table S1 by UMI status. The average Framingham risk score for coronary heart disease was 11.2% (standard deviation 10.2%) in the participants with UMI and 8.4% (standard deviation 8.3%) in the participants without UMI.

**Table 1:** Characteristics considered in the basic assessment tool by unrecognized myocardial infarction status

	**UMI**	**No MI**	**p-value**
	**(n = 740)**	**(n = 15,913)**	
Age (years)	66.7 (9.9)	63.2 (9.6)	<0.001
Female (%)	61.8	65.8	0.03
African-American (%)	42.2	41.3	0.66
Smoking status (%)			0.003
Never smoker	45.1	50.0	
Past smoker	36.8	35.9	
Current smoker	18.1	14.1	
Alcohol use (%)			0.40
None	64.0	62.6	
Moderate	32.8	33.3	
Heavy	3.2	4.1	
History of stroke (%)	6.8	4.3	0.002
History of transient ischemic attack (%)	4.9	3.2	0.01
History of deep vein thrombosis (%)	4.6	4.6	0.93
History of peripheral vascular disease (%)	1.6	1.1	0.24
History of dialysis (%)	0.4	0.2	0.17
History of falls (%)	18.7	15.5	0.02
Self-reported diabetes (%)	24.6	18.9	<0.001
Self-reported hypertension (%)	68.0	54.4	<0.001
Self-reported dyslipidemia (%)	50.1	49.3	0.64
Family history of myocardial infarction (%)	31.1	33.7	0.13
Systolic blood pressure (mmHg)	130 (18)	126 (16)	<0.001
Diastolic blood pressure (mmHg)	77 (10)	76 (10)	0.12
Current use of antihypertensives (%)	64.0	49.5	<0.001
Body mass index (kg/m^2^)	29.0 (6.9)	29.4 (6.3)	0.10

Numbers in table are mean (standard deviation) or percent. *UMI* Unrecognized myocardial infarction; *MI* Myocardial infarction.

### Basic assessment tool

**Figure 1:**
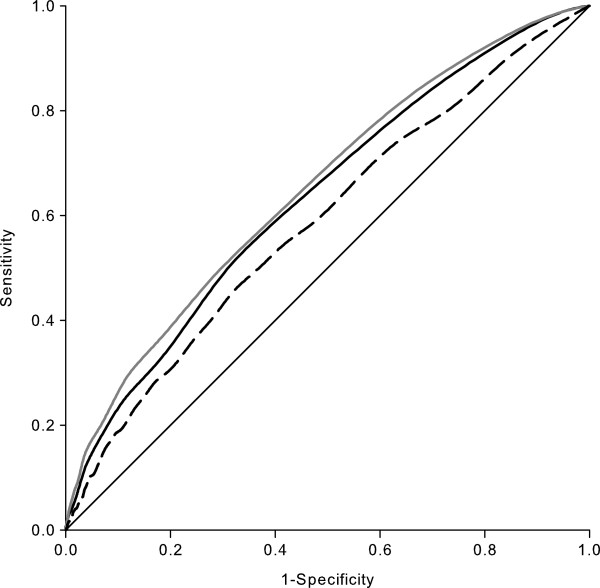
**Receiver operating characteristic curves for the unrecognized myocardial infarction assessment tools.** Basic assessment tool (solid black line), expanded assessment tool (solid gray line), and Framingham risk score for coronary heart disease (dashed black line).

**Figure 2:**
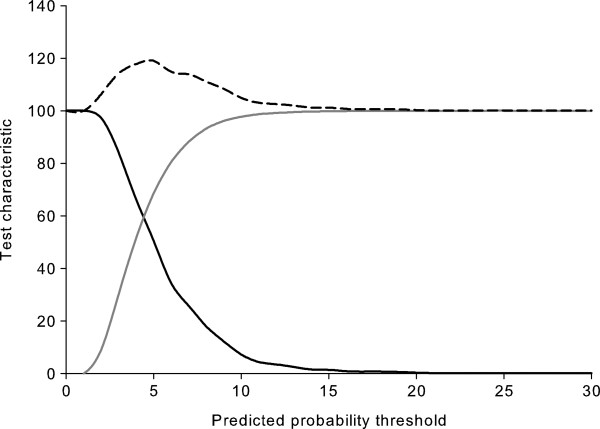
**Test characteristics of the unrecognized myocardial infarction basic assessment tool.** Sensitivity (solid black line), specificity (solid gray line), and sum of sensitivity and specificity (dashed black line).

Established MI risk factors such as age, male sex, cigarette smoking, diabetes, and hypertension were associated with UMI in unadjusted analyses (Table 2). Age discriminated between those who did and did not have UMI better than any other potential correlate, followed by current use of antihypertensive medications, self-reported hypertension, and systolic blood pressure. The model with all of the potential correlates of UMI listed in Table 2 had a c-statistic of 0.646 (95% CI 0.621-0.672). The backward selection procedure resulted in an assessment tool with 11 items: age, race, smoking status, body mass index (linear and squared terms), systolic blood pressure, and self-reported history of transient ischemic attack, deep vein thrombosis, falls, diabetes, and hypertension. None of the omitted variables improved the c-statistic by more than 0.005 when they were added back to the model. The c-statistic for the basic assessment tool was 0.638 (95% CI 0.617 - 0.659, p < 0.001 for test of no discrimination) (Figure 1). In order to detect at least 80% of individuals with UMI using the basic assessment tool, everyone with a predicted probability > 3% would need to undergo ECG screening. In the REGARDS study population, 70% of participants had a > 3% predicted probability. The > 3% predicted risk threshold corresponded to a specificity of 30%. A predicted probability of UMI of 5% maximized the sum of sensitivity and specificity and corresponded to a sensitivity of 50% and a specificity of 69%; 32% of REGARDS participants had a predicted probability > 5% (Figure 2). Results were similar in subgroups defined by age (< 65 years or ≥ 65 year), sex, and race.

**Table 2:** Odds ratios and c-statistics for unrecognized myocardial infarction in the basic assessment tool

	**Univariate c-statistic**	**Odds ratio (95% CI)**	**Odds ratio (95% CI)**
		**Unadjusted**	Backwards selection^*^
Age (10 years)	0.602	1.45 (1.35-1.57)	1.38 (1.27-1.49)
Sex	0.520		
Male		1.00 (reference)	--
Female		0.84 (0.72-0.98)	--
Race	0.504		
White		1.00 (reference)	1.00 (reference)
African-American		1.03 (0.89-1.20)	0.93 (0.79-1.09)
Smoking status	0.531		
Never smoker		1.00 (reference)	1.00 (reference)
Past smoker		1.13 (0.96-1.33)	1.08 (0.92-1.28)
Current smoker		1.42 (1.16-1.75)	1.60 (1.29-1.98)
Alcohol use	0.509		
None		1.00 (reference)	--
Moderate		0.97 (0.82-1.13)	--
Heavy		0.75 (0.49-1.15)	--
History of stroke	0.512	1.60 (1.19-2.16)	--
History of transient ischemic attack	0.509	1.58 (1.11-2.26)	1.18 (0.82-1.69)
History of deep vein thrombosis	0.500	1.02 (0.72-1.45)	0.89 (0.63-1.28)
History of peripheral vascular disease	0.502	1.43 (0.79-2.57)	--
History of dialysis	0.501	2.24 (0.68-7.36)	--
History of falls	0.516	1.24 (1.03-1.51)	1.15 (0.95-1.40)
Self-reported diabetes	0.528	1.40 (1.18-1.66)	1.25 (1.04-1.50)
Self-reported hypertension	0.568	1.78 (1.52-2.09)	1.56 (1.32-1.86)
Self-reported dyslipidemia	0.504	1.04 (0.89-1.20)	--
Family history of myocardial infarction	0.513	0.89 (0.76-1.04)	--
Systolic blood pressure (10 mmHg)	0.564	1.15 (1.10-1.19)	1.08 (1.03-1.13)
Diastolic blood pressure (10 mmHg)	0.510	1.06 (0.98-1.15)	--
Current use of antihypertensives	0.572	1.81 (1.55-2.12)	--
Body mass index (5 kg/m^2^)	0.524	0.95 (0.90-1.01)	0.89 (0.83-0.96)
Body mass index squared (5[kg/m^2^]^2^)	0.521	1.004 (1.001-1.007)	1.008 (1.003-1.012)

^*^ C statistic for backward selection model = 0.638 (95% CI 0.617-0.659).

### Expanded assessment tool

Unadjusted associations between the expanded set of covariates and presence of UMI are presented in Additional file 3: Table S2. The model with all of the expanded set of potential correlates of UMI had a c-statistic of 0.677 (95% CI 0.650-0.704). The backward selection procedure produced an expanded assessment tool with 15 items (age, race, income, smoking status, self-reported hypertension, unrecognized dyslipidemia, perceived stress score, waking at night due to breathlessness, body mass index and body mass index squared, HDL cholesterol squared, systolic blood pressure, albumin to creatinine ratio, heart rate, and heart rate squared) and a c-statistic of 0.654 (95% CI 0.634-0.674, p < 0.001 for test of no discrimination) (Figure 1). To obtain a sensitivity of 80%, a > 3% predicted probability threshold would be needed, which corresponded to a specificity of 34%.

### Comparison with the Framingham risk score for coronary heart disease

The c-statistic for identifying participants with UMI using the Framingham risk score for coronary heart disease was 0.587 (95% CI 0.566-0.609) (Figure 1). If participants considered high risk of coronary heart disease (Framingham risk score of 20% or above) were screened, 17% of UMIs would be detected (sensitivity) while 91% of those without UMI would not receive ECG (specificity).

## Discussion

In the current study, we attempted to develop tools for identifying individuals with an elevated probability of UMI as determined by ECG among those without a self-reported history of MI. Such a tool would allow targeting of ECG screening for UMI to higher-risk individuals. However, neither the tool built using potential correlates routinely collected nor the expanded tool which considered a large battery of variables had a c-statistic greater than 0.7, a widely used guideline for adequate discrimination [14]. Furthermore, the test characteristics were such that a reasonably high sensitivity required a very low specificity.

Previous studies have found that coronary heart disease risk factors such as older age, male sex, diabetes, hypertension, dyslipidemia, and smoking are associated with UMI [15]. While the traditional coronary heart disease risk factors were associated with prevalent UMI in the REGARDS study population, they did not discriminate well between participants with and without UMI. For incident coronary heart disease, the Framingham risk score, which incorporates many of the risk factors and is recommended for use in the general United States population without known cardiovascular disease, has been shown to have a c-statistic greater than 0.7 in many, but not all, populations [16]. In the current study, the c-statistic for UMI associated with the Framingham risk score was 0.587. The Framingham Risk Score is a validated risk score for incident coronary heart disease. Its use for prevalent UMI is unvalidated. The AUC of FRS in lower than expected but this should be interpreted with caution. The difference in the ability to discriminate between people who will develop coronary heart disease and those who will not and the ability to discriminate between people with prevalent UMI and those without prevalent UMI may be because of the variety of processes that are involved in having UMI. An ischemic injury causing Q-waves must occur but go unrecognized by the individual or the individual’s health care providers or both. Then factors related to survival with MI come into play in order to become a member of the REGARDS study. Finally, ECG abnormalities are known to resolve over time in some people [17]; the participants in this study considered to have UMI were only those with persistent ECG indications of MI. The complicated pathway to UMI may hinder identification of populations with high prevalence of UMI.

The current study focused on the c-statistic as the metric of discrimination for the UMI assessment tools. C-statistics are known to be higher in the population in which they are derived than in independent populations, and therefore it is customary to validate the assessment tools in external populations. In the current study, this step was not taken because of the poor performance of the tools in the derivation cohort. The difference in c-statistics between the UMI assessment tools, which were developed in the REGARDS study, and the Framingham risk score for coronary heart disease would likely have been smaller in an independent population. We did not examine calibration metrics in this study because, in the absence of acceptable discrimination, even a very well-calibrated assessment tool would not add important information to the decision of whether or not to screen for UMI.

The major strengths of this study include the large, diverse REGARDS study population and the wide variety of potential correlates which have been measured. There are also limitations of this study. We used ECG criteria for defining UMI, though Q-waves on ECG are absent in some patients with documented MI [17], may be due to conditions other than MI [18,19], and do not agree well with myocardial scars detected on cardiac magnetic resonance imaging with gadolinium contrast [19]. The inability to detect UMI not associated with Q-waves is an inherent limitation of the ECG method for detection of UMI; however, the use of the MC scheme reduces the concern about Q-waves due to other conditions which take precedence over MI in the coding system [12]. We believe that ECG would be more appropriate for screening in a general population than cardiac magnetic resonance imaging with gadolinium contrast because of the greater availability, lower cost, and time needed for ECGs, and the potential for serious adverse reactions to gadolinium contrast.

We relied on self-report to identify individuals with recognized MI and history of coronary heart disease. Previous studies have shown that some participants report MI when there is no clinical record of MI, and others may report no history of MI when a diagnosis of MI has been documented [20,21]. Some of the participants in the REGARDS study population who we considered to have UMI may have a known MI from the perspective of their healthcare providers. While the patient-centered approach we took was designed to simulate patient encounters with physicians, it is possible that lack of accuracy in self-report of MI makes an UMI assessment tool impractical in the clinical setting. Many of the potential correlates of UMI were also self-reported. Some of the correlates are by definition self-reported, such as self-rated health, perceived stress, and depression symptoms, but different approaches may be more reliable for other covariates, such as prior medical history. In these cases, reliance on self-reported data could lead to increased error and an underestimation of the ability of these factors to discriminate between individuals with and without UMI. Finally, ECG, blood pressure, and biomarkers were measured during a single in-home visit and are subject to measurement error which could also lead to underestimation of the ability to discriminate between individuals with and without UMI. However, these measurements were made using a research protocol, which may be more reliable than measures obtained in routine clinical practice; this may result in worse performance of the tool in clinical care settings than we report here.

## Conclusions

Although the assessment tools for UMI performed significantly better than chance, they demonstrated low levels of discrimination. Given the test properties of the assessment tools, they are unlikely to be useful in clinical practice. These data suggest that it will be difficult to define a population with a high prevalence of UMI for targeted ECG screening.

## Abbreviations

CESD-4: 4-Item center for epidemiologic studies depression scale; CKD-EPI: Chronic kidney disease epidemiology collaboration; ECG: Electrocardiogram; eGFR: Estimated glomerular filtration rate; HDL: High density lipoprotein; LDL: Low density lipoprotein; MC: Minnesota code; MI: Myocardial infarction; REGARDS: REasons for geographic and racial differences in stroke; SF-12: Medical outcomes study short form-12; UMI: Unrecognized myocardial infarction.

## Competing interests

The authors declare that they have no competing interests.

## Authors’ contributions

EBL lead the design of the study, conducted the statistical analysis, and drafted the manuscript. MMS contributed to the study design, collected the data, and critically revised the manuscript. MLK contributed to the study design and critically revised the manuscript. EZS contributed to the study design, collected the data, and critically revised the manuscript. SPG contributed to the study design, collected the data, and critically revised the manuscript. SEJ contributed to the study design, collected the data, and critically revised the manuscript. PM contributed to the study design, advised on the statistical analysis, and critically revised the manuscript. All authors read and approved the final manuscript.

## Pre-publication history

The pre-publication history for this paper can be accessed here:

http://www.biomedcentral.com/1471-2261/13/23/prepub

## Supplementary Material

Additional file 1 Reasons for geographic and racial differences in stroke baseline data collection forms. Click here for file

Additional file 2: Table S1 Characteristics considered in the expanded assessment tool by unrecognized myocardial infarction status. Click here for file

Additional file 3: Table S2 Odds ratios and c-statistics for unrecognized myocardial infarction in the expanded assessment tool. Click here for file
