# Supplementary material for: Assessment tools for unrecognized myocardial infarction: a cross-sectional analysis of the REasons for geographic and racial differences in stroke population
Source: BMC Cardiovasc Disord. 2013 Mar 26;13:23. doi: 10.1186/1471-2261-13-23 (PMC3617994; doi:10.1186/1471-2261-13-23)
Supplement: Additional file 1 — Reasons for geographic and racial differences in stroke baseline data collection forms. [file 1471-2261-13-23-S1.doc]

**CATI Baseline Survey Codebook**

R·E·G·A·R·D·S

Survey Research Unit

University of Alabama at Birmingham

Updated: 8/17/05

REGARDS

CATI Baseline Survey Codebook

Updated: 11/18/04

**OPENING 1: Introduction**

INTRODUCTION AND RESPONDENT SELECTION REVISED EFFECTIVE 6/9/04 TO

INCORPORATE HOUSEHOLD ENUMERATION AND SELECTION OF RESPONDENT FROM ALL AGE ELIGIBLE RESPONDENTS. IF A SELECTED RESPONDENT IS FOUND TO BE INELIGIBLE BECAUSE OF RACE, HEALTH ISSUES OR ON A LIST TO ENTER A NURSING HOME, ANOTHER RESPONDENT, IF AVAILABLE, IS SELECTED.

Q: Intro ++++++++++++++++++++++++++++++++++++++++

INTERVIEWER: Please dial number.

Hello! My name is ___________________ and I'm calling long distance

from the University of Alabama at Birmingham. About a week ago we sent

a letter to the family of <INSERT NAME> to tell you about the REGARDS stroke project and to let you know that would be calling to invidte one member of your household to participate in this important project.

Have I reached the <INSERT NAME> family at <INSERT PHONE NUMBER>?

1. Correct family Proceed to NumAdult

2. Wrong number for family Thank and terminate

Q: NumAdult

As I mentioned, I am calling for the REGARDS stroke project. This is a nationwide study designed to understand why people in different parts of the country and people of different ethnic groups have different rates of stroke. This project is designed to help understand the risk factors for stroke.

I’d like to ask you just a couple of questions to see if you or someone in your family might qualify for this survey.

How many members of your family are over the age of 18? ______________

Q: NumSrs

And of those <INSERT # from NumAdult> adults, how many are age 45 or older? _______

IF NumSrs = 0 Thank and terminate

IF NumSrs = 1 Skip to Intro2

IF NumSrs > 1 Skip to QAges

Q: QAges

Starting with the oldest adult age 45 or older, can you please give me the ages of all the adults age 45 or older?

CATI then selects one adult from list of adults age 45 or older to be interviewed.

Proceed to QSelect

Q: QSelect

May I speak with the person in your household who is <INSERT AGE OF SELECTED RESPONDENT>?

Q: INTRO2 +++++++++++++++++++++++++++++++++

Once correct respondent is on the phone and interviewer has introduced himself/herself:

REGARDS is a nationwide project designed to understand why people in different parts of the country and people of different ethnic groups have different rates of stroke. Stroke is the 3rd leading cause of death in our country and kills more people each year than breast cancer and AIDS combined. It is also the number one cause of disability. This project is designed to help understand the risk factors for stroke. To do this, we need to recruit 30,000 healthy volunteers to take part. We are calling today to invite you to be one of these volunteers. This will be an important first step in finding ways to prevent strokes in the future.

Before I give you any more details, I would like to ask just a few questions to make sure you are eligible to participate.

INTERVIEWER: Press any key to continue

**OPENING 2: Demographics I**

Q: QDOB ++++++++++++++++++++++++++++++++++++++++

What is your date of birth?

_ _-_ _ -_ _ _ _

01‑01‑1888 Don't Know/Not Sure

01‑01‑1889 Refused

INTERVIEWER: Make sure respondent is 45 years of age or older

:

C: Range Check for Age 45 or older

IF (ANS = 18880101) DISPOS = 33

IF (ANS = 18890101) DISPOS = 33

IF (ANS = 18870101) DISPOS = 33

RESPAGE = Datedif (QDOB Sysdate)

RESPAGE = RESPAGE/365

TRUEAGE = RESPAGE

CMDO RESPAGE "Age" 1

CMDO TRUEAGE "Trueage" 1

IF (RESPAGE < 45) SKP Sorry1

IF (RESPAGE = 45) SKP QLatino

IF (RESPAGE > 45) SKP QLatino

Eligibility age changed 4/23/04 from age 55 to age 45

Beginning 6/9/04: If respondent is under age 45, return to QAges and select another portential respondent from household and proceed. If there are no other age eligible respondents, thank and terminate.

Q: Sorry1 ++++++++++++++++++++++++++++++++++++++++

Thank you very much for the time you've spent with me on the phone, but to participate in this study you must be 45 years of age or older.

INTERVIEWER: Press any key to continue

DISPOS = 33 CTRLEND

Q: QLatino ++++++++++++++++++++++++++++++++++++++++

Are you Hispanic or Latino?

1. Yes

2. No

8. Don't Know/Not Sure

9. Refused

NOTE: Only white or black, non‑hispanic eligible

Beginning 6/9/04, if respondent is Latino, return to QAges question and select another potential respondent from household and proceed. If there are no other age eligible respondents, thank and terminate.

IF (ANS <> 2) SKP SORRY2

IF (ANS = 2) SKP QRace

Q: QRace ++++++++++++++++++++++++++++++++++++++++

What is your race? Would you say White, Black or African American, Asian, Native Hawaiian or Other Pacific Islander American Indian, Alaska Native or some other race?

1. White

2. Black or African American

3. Asian

4. Native Hawaiian or Other Pacific Islander

5. American Indian, Alaska Native or

6. Other Specify:

8. Don't Know/Not Sure

9. Refused

Beginning 6/9/04, if respondent is not either White or Black/African American, return to QAges and select another potential respondent from household and proceed. If there are no other age eligible respondents, thank and terminate.

IF (ANS = 1) TrueRace = "W"

IF (ANS = 2) TrueRace = "B"

CMDO TrueRace "TrueRace" 1

IF (ANS > 2) SKP SORRY2

IF (ANS < 3) SKP QGender

Q: Sorry2 ++++++++++++++++++++++++++++++++++++++++

Thank you for taking a few minutes to speak with me, but for the purposes of this study we are trying to interview equal numbers of different racial and ethnic groups and have already met our quota in your area. Thank you again, and have a great day.

INTERVIEWER: Press any key to end interview

DISPOS = 34 CTRLEND

Q: QGender ++++++++++++++++++++++++++++++++++++++++

INTERVIEWER: ENTER SEX OF RESPONDENT (Ask only if necessary.)

1. Male

2. Female

IF (ANS = 1) GENDER = "M"

IF (ANS = 2) GENDER = "F"

CMDO GENDER "TrueGender" 1

**OPENING 3: Health Related Eligibility**

Q: QNursing ++++++++++++++++++++++++++++++++++++++++

Are you currently on a waiting list for a nursing home?

1. Yes

2. No

8. Don't Know/Not Sure

9. Refused

Beginning 6/9/04, if respondent is currently on a waiting list for a nursing home, return to QAges and select another potential respondent from household and proceed. If there are no other age eligible respondents, thank and terminate.

IF (ANS <> 2) SKP SORRY6

IF (ANS = 2) SKP QCancer

Q: Sorry6 ++++++++++++++++++++++++++++++++++++++++

Thank you so much for your time, but participation in this project requires that you live independently and have no plans for entering a nursing home or other facility during the follow up period. I hope you have a nice day.

INTERVIEWER: Press any key to terminate survey

DISPOS = 38 CTRLEND

Q: QCancer ++++++++++++++++++++++++++++++++++++++++

Have you ever been diagnosed with cancer?

1. Yes

2. No

8. Don't Know/Not Sure

9. Refused

IF (ANS > 1) SKP QMedical

Q: QCancerb ++++++++++++++++++++++++++++++++++++++++

Have you been treated with chemotherapy or radiation in the past two years?

1. Yes

2. No

8. Don't Know/Not Sure

9. Refused

If respondent has been treated with chemotherapy or radiation in the past two years or answers DK/NS to this question, return to QAges and select another potential respondent from household and proceed. If there are no other age eligible respondents, thank and terminate.

IF (ANS <> 2) SKP Sorry3

Q: QMedical ++++++++++++++++++++++++++++++++++++++++

Do you have any serious medical conditions that would prevent you from doing a 5 minute survey twice a year for the next four years?

1. Yes

2. No

8. Don't Know/Not Sure

9. Refused

Beginning 6/9/04: if respondent reports that he/she does have a medical condition that would prevent him/her from completing a 5 minute survey twice a year for the next four years, or respondends with DK or Refused, return to QAges and select another potential respondent from household and proceed. If there are no additional eligible respondents, thank and terminate.

IF (ANS <> 2) SKP SORRY3

IF (ANS = 2) SKP QELIG1

Q: Sorry3 ++++++++++++++++++++++++++++++++++++++++

Thank you so much for your time, but we will not be able to enrol you as a volunteer in this project. Thanks for your time and interest. I hope you have a very nice day.

INTERVIEWER: Press any key to terminate interview.

DISPOS = 35 CTRLEND

Q: QELIG1 ++++++++++++++++++++++++++++++++++++++++

Great! You are eligible to participate. Let me give you a few more details about this project, then I'll give you the chance to ask me some questions.

Scientists do not yet understand who is at risk for stroke and why. We do know, however, that strokes occur more often in some parts of the country and among different racial groups. But at this time, those differences are hard to explain. This project will help scientists understand why these differences exist.

INTERVIEWER: Press any key to continue

CMDO SYSDATE "Intdate" 1

Q: QELIG2 ++++++++++++++++++++++++++++++++++++++++

Participating is easy! First you and I will do a telephone survey that takes about 30 minutes to get some information about your general health.

Next, a health professional will come to your home to measure your height, weight, hip, waist and blood pressure. He or she will also take a blood sample, a urine sample and an electrical recording of your heart. This in home exam will take about 60 minutes. The health professional will also give you other forms to complete on your own and return by mail. We will mail you a report about your test with results and $30 after the in‑home visit to reimburse you for your time.

You can schedule this in-home exam at your home, at a friend’s home, at work or some place else of your choosing. However, this in-home exam will need to be scheduled for a morning time, Monday through Thursday before noon. We could even come before you go to work if that is most convenient for you. Is this something you think you could schedule?

1. Yes
2. No

Beginning 6/9/04: if respondent is not able to schedule an in home exam Monday through Thursday before noon, return to QAges question and select another potential respondent from the household and proceed. If there are no other age eligible respondents, thank and terminate.

Q: QELIG3 ++++++++++++++++++++++++++++++++++++++++

The only other part of the project involves us calling you twice a year to see how you have been doing. If you have been hospitalized or had any possible symptoms of stroke since your last call, we will request copies of your medical records.

Do you have any questions I can answer for you?

INTERVIEWER: See FAQ sheet in the black binder for answers to frequently asked questions

Press any key to continue

Q: QAGREE ++++++++++++++++++++++++++++++++++++++++

Do you agree to participate in the REGARDS project?

1. Yes

2. No

IF (ANS = 1) SKP Qstart

IF (ANS = 2) SKP thanks

Q: thanks ++++++++++++++++++++++++++++++++++++++++

INTERVIEWER: Ask respondent to tell you why he or she is not interested in participating in the study. If possible, correct any misunderstandings and answer objections using the FAQ sheet

in the black binder.

1. Interested in participaing

2. Still not interested

IF (ANS = 1) SKP Qstart

IF (ANS = 2) SKP thank2

Q: thank2 ++++++++++++++++++++++++++++++++++++++++

Well, thank you for taking the time for briefly speaking with me today. If you change your mind about participating in this important study, please call toll free 1‑866‑463‑6667. Have a very nice day.

INTERVIEWER: Press any key to continue

DISPOS = 09 CTRLEND

Q: QStart ++++++++++++++++++++++++++++++++++++++++

Great! Let's get started. All your answers will be kept totally confidential, and you don't have to answer any questions you don't want to. There are no right or wrong answers and if you can't remember some of the information I'll be asking for, just let me know and we'll move on.

INTERVIEWER: Press any key to continue

Q: QName

Let me start by getting your first and last names:

First Name ______________________________

Last Name _________________________________

**SECTION 1: Stroke‑Free Phenotype**

Q: Q1_1 ++++++++++++++++++++++++++++++++++++++++

The first set of questions asks about whether you have had a stroke or a mini‑stroke.

Were you ever told by a physician that you had a stroke?

1. Yes

2. No

8. Don't Know/Not Sure

9. Refused

IF (ANS <> 1) SKP Q1_2

Q: Q1_1a ++++++++++++++++++++++++++++++++++++++++

How many strokes have you had?

_ _ _ Enter number of strokes

888 Don't Know/Not Sure

999 Refused

IF (ANS > 10 AND < 888) REASK

Q: Q1_1b ++++++++++++++++++++++++++++++++++++++++

IF (Q1_1a = 1) How old were you when you had your stroke?

IF (Q1_1a > 1) How old were you when you had your first stroke?

_ _ _ Enter age in years

If not sure, ask for best guess. If not possible to guess exact age use:

770 Less than 10 years old

771 Between 10 and 19

772 Between 20 and 29

773 Between 30 and 39

774 Between 40 and 49

775 Between 50 and 59

776 Between 60 and 69

777 Between 70 and 79

778 Between 80 and 89

779 Between 90 and 99

780 Over 99

888 Don't Know/Not Sure

999 Refused

IF (ANS > RESPAGE) AND IF (ANS < 769) REASK

IF (Q1_1a = 1) SKP Q1_2

Q: Q1_1c ++++++++++++++++++++++++++++++++++++++++

How old were you when you had your last stroke?

_ _ _ Enter age in years

If not sure, ask for best guess. If not possible to guess exact age use:

770 Less than 10 years old

771 Between 10 and 19

772 Between 20 and 29

773 Between 30 and 39

774 Between 40 and 49

775 Between 50 and 59

776 Between 60 and 69

777 Between 70 and 79

778 Between 80 and 89

779 Between 90 and 99

780 Over 99

888 Don't Know/Not Sure

999 Refused

IF (ANS > RESPAGE) AND IF (ANS < 769) REASK

SKP Q2_1

Q: Q1_2 ++++++++++++++++++++++++++++++++++++++++

Were you ever told by a physician that you had a mini‑stroke or TIA, also known as a transient

ischemic attack?

1. Yes

2. No

8. Don't Know/Not Sure

1. Refused

IF (Q1_1 = 1) SKP Q2_1

Q: Q1_3 ++++++++++++++++++++++++++++++++++++++++

Have you ever had sudden painless weakness on one side of your body?

1. Yes

2. No

8. Don't Know/Not Sure

9. Refused

Q: Q1_4 ++++++++++++++++++++++++++++++++++++++++

Have you ever had sudden numbness or a dead feeling on one side of your body?

1. Yes

2. No

8. Don't Know/Not Sure

9. Refused

Q: Q1_5 ++++++++++++++++++++++++++++++++++++++++

Have you ever had sudden painless loss of vision in one or both eyes?

1. Yes

2. No

8. Don't Know/Not Sure

9. Refused

Q: Q1_6 ++++++++++++++++++++++++++++++++++++++++

Have you ever suddenly lost one half of your vision?

1. Yes

2. No

8. Don't Know/Not Sure

9. Refused

Q: Q1_7 ++++++++++++++++++++++++++++++++++++++++

Have you ever suddenly lost the ability to understand what people were saying?

1. Yes

2. No

8. Don't Know/Not Sure

9. Refused

Q: Q1_8 ++++++++++++++++++++++++++++++++++++++++

Have you ever suddenly lost the ability to express yourself verbally or in writing?

1. Yes

2. No

8. Don't Know/Not Sure

9. Refused

**SECTION 2: Medical History**

Q: Q2_1 ++++++++++++++++++++++++++++++++++++++++

These next questions ask about your medical history.

Has a doctor or other health professional ever told you that you had diabetes or high blood sugar?

1. Yes

2. No

8. Don't Know/Not Sure

9. Refused

IF (ANS > 1) SKP Q2_1b3

IF (ANS = 1) AND (GENDER = "M") SKP Q2_1b

Q: Q2_1a ++++++++++++++++++++++++++++++++++++++++

Did you have diabetes only while you were pregnant?

1. Yes

2. No

8. Don't Know/Not Sure

9. Refused

IF (ANS = 1) SKP Q2_2

Q: Q2_1b ++++++++++++++++++++++++++++++++++++++++

Are you taking medicine for diabetes?

1. Yes

2. No

8. Don't Know/Not Sure

9. Refused

IF (ANS > 1) SKP Q2_1b3

Q: Q2_1b1 ++++++++++++++++++++++++++++++++++++++++

Do you take pills?

1. Yes

2. No

8. Don't Know/Not Sure

9. Refused

Q: Q2_1b2 ++++++++++++++++++++++++++++++++++++++++

Do you take insulin?

1. Yes

2. No

8. Don't Know/Not Sure

9. Refused

Q: Q2_1b3 ++++++++++++++++++++++++++++++++++++++++

Has a doctor or other health professional ever told you that you had kidney failure?

1. Yes

2. No

8. Don't Know/Not Sure

9. Refused

IF (ANS > 1) SKP Q2_1b5

Question revised 4/20/04 changing kidney failure to “kidney disease”

And adding Questions Q2_1b5 and Q2_1b6 and changing skip pattern

From Q2_2 to Q2_1b5

Q: Q2_1b4 ++++++++++++++++++++++++++++++++++++++++

Are you on dialysis?

1. Yes

2. No

8. Don't Know/Not Sure

9. Refused

Q: Q2_1b5 +++++++++++++++++++++++++++++++++++++++++++++++++

Has any one of your immediate family ever been told that he or she had kidney failure? This would be someone who is on or had been or dialysis or someone who had a kidney transplant.

1. Yes

2. No

8. Don’t Know/Not Sure

9. Refused

INTERVIEWER: Immediate family is defined as first degree relatives like a

parent, a brother or sister or a child.

IF (ANS > 1) SKP Q2_2

Q: Q2_1b6 +++++++++++++++++++++++++++++++++++++++++++++++++++++++

What relative or relatives had or has kidney failure?

INTERVIEWER: Do not read responses. Mark all that apply

1. Parent
2. Grandparent
3. Aunt or Uncle
4. Sibling (Brother or Sister)
5. Child
6. Cousin
7. Non-Blood relative (like a step parent or god parent)
8. Other: Please Specify__________________________
9. Don’t Know/Not Sure
10. Refused

Q: Q2_2 ++++++++++++++++++++++++++++++++++++++++

Has a doctor or other health professional ever told you that you had a myocardial infarction or heart attack?

1. Yes

2. No

8. Don't Know/Not Sure

9. Refused

IF (ANS > 1) SKP Q2_2d

Q: Q2_2a ++++++++++++++++++++++++++++++++++++++++

How many myocardial infarctions or heart attacks have you had?

_ _ _ Enter number of heart attacks

666 More than 10

888 Don't Know/Not Sure

999 Refused

IF (ANS > 10) AND IF (ANS < 888) REASK

Note: Response option: 666 “More than 10” added 6/4/04

Q: Q2_2b ++++++++++++++++++++++++++++++++++++++++

How old were you when you had your/your first heart attack or myocardial infarction?

_ _ _ Enter age in years

If not sure, ask for best guess. If not possible to guess exact age use:

770 Less than 10 years old

771 Between 10 and 19

772 Between 20 and 29

773 Between 30 and 39

774 Between 40 and 49

775 Between 50 and 59

776 Between 60 and 69

777 Between 70 and 79

778 Between 80 and 89

779 Between 90 and 99

780 Over 99

888 Don't Know/Not Sure

999 Refused

IF (ANS > RESPAGE) AND IF (ANS < 769) REASK

IF (Q2_2a = 1) SKP Q2_2d

Q: Q2_2c ++++++++++++++++++++++++++++++++++++++++

How old were you when you had your last heart attack or myocardial infarction?

_ _ _ Enter age in years

If not sure, ask for best guess. If not possible to guess exact age use:

770 Less than 10 years old

771 Between 10 and 19

772 Between 20 and 29

773 Between 30 and 39

774 Between 40 and 49

775 Between 50 and 59

776 Between 60 and 69

777 Between 70 and 79

778 Between 80 and 89

779 Between 90 and 99

780 Over 99

888 Don't Know/Not Sure

999 Refused

IF (ANS > RESPAGE) AND IF (ANS < 769) REASK

Q: Q2_2d ++++++++++++++++++++++++++++++++++++++++

Has a doctor or other health professional ever told you that you had atrial fibrillation?

1. Yes

2. No

8. Don't Know/Not Sure

9. Refused

Q: Q2_2e ++++++++++++++++++++++++++++++++++++++++

Do you ever have to sleep on two or more pillows to help you breathe?

1. Yes

2. No

8. Don't Know/Not Sure

9. Refused

Q: Q2_2f ++++++++++++++++++++++++++++++++++++++++

Do you ever wake at night because you are having trouble breathing?

1. Yes

2. No

8. Don't Know/Not Sure

9. Refused

Q: Q2_2g ++++++++++++++++++++++++++++++++++++++++

Has a doctor ever told you that you had pulmonary embolus or blood clots in your lungs?

1. Yes

2. No

8. Don't Know/Not Sure

9. Refused

IF (ANS > 1) SKP Q2_2i

Q: Q2_2h ++++++++++++++++++++++++++++++++++++++++

Were you treated with blood thinning medication like warfarin, coumadin or heparin for it?

1. Yes

2. No

8. Don't Know/Not Sure

9. Refused

Q: Q2_2i ++++++++++++++++++++++++++++++++++++++++

Has a doctor ever told you that you had phlebitis, deep venous thrombosis or blood clots in your legs?

1. Yes

2. No

8. Don't Know/Not Sure

9. Refused

IF (ANS > 1) SKP Q2_3

Q: Q2_2j ++++++++++++++++++++++++++++++++++++++++

Were you treated with blood thinning medication like warfarin, coumadin or heparin for it?

1. Yes

2. No

8. Don't Know/Not Sure

9. Refused

Q: Q2_3 ++++++++++++++++++++++++++++++++++++++++

Has a doctor or other health professional ever told you that you have high blood pressure?

1. Yes

2. No

8. Don't Know/Not Sure

9. Refused

IF (ANS > 1) SKP Q2_4

IF (ANS = 1) AND IF (GENDER ="M") SKP Q2_3a

Q: Q2_3preg ++++++++++++++++++++++++++++++++++++++++

Was this only when you were pregnant?

1. Yes

2. No

8. Don't Know/Not Sure

9. Refused

IF (ANS <> 2) SKP Q2_4

Q: Q2_3a ++++++++++++++++++++++++++++++++++++++++

How old were you when you were first told that you have high blood pressure?

_ _ _ Enter age in years

If not sure, ask for best guess. If not possible to guess exact age use:

770 Less than 10 years old

771 Between 10 and 19

772 Between 20 and 29

773 Between 30 and 39

774 Between 40 and 49

775 Between 50 and 59

776 Between 60 and 69

777 Between 70 and 79

778 Between 80 and 89

779 Between 90 and 99

780 Over 99

888 Don't Know/Not Sure

999 Refused

IF (ANS > RESPAGE) AND IF (ANS < 769) REASK

Q: Q2_3b ++++++++++++++++++++++++++++++++++++++++

Have you ever taken medicine for high blood pressure? This could be pills or other medicines.

1. Yes

2. No

8. Don't Know/Not Sure

9. Refused

IF (ANS > 1) SKP Q2_4

Q: Q2_3c ++++++++++++++++++++++++++++++++++++++++

Are you NOW taking any medicine for high blood pressure?

1. Yes

2. No

8. Don't Know/Not Sure

9. Refused

Q: Q2_4 ++++++++++++++++++++++++++++++++++++++++

Have you ever been told by a doctor that you have high cholesterol or an abnormal level of fats in your blood?

1. Yes

2. No

8. Don't Know/Not Sure

9. Refused

IF (ANS > 1) SKP Q2_5

Q: Q2_4a ++++++++++++++++++++++++++++++++++++++++

Are you now taking any medicine to treat it?

1. Yes

2. No

8. Don't Know/Not Sure

9. Refused

INTERVIEWER: If respondent is unsure, you may list some of the common medications

Zocor (generic = simvastatin) Lipitor (generic = atorvastatin)

Lopid (generic = gemfibrozil) Pravachol (generic = pravastatin)

Zetia (generic = ezetimibe) Niaspan (generic = niacin)

Interviewer instructions listing some of the most commonly used medications added 8/9/04

Q: Q2_5 ++++++++++++++++++++++++++++++++++++++++

Approximately how many times have you been treated with antibiotics in the past year? If you don't remember the exact number, please give us your best guess.

_ _ _ Number of times

888 Don't Know/Not Sure

999 Refused

Q: Q2_6 ++++++++++++++++++++++++++++++++++++++++

Have you lost any of your teeth due to gum disease?

1. Yes

2. No

8. Don't Know/Not Sure

9. Refused

IF (ANS > 1) SKP Q3_1

Q: Q2_6a ++++++++++++++++++++++++++++++++++++++++

How many teeth have you lost due to gum disease?

_ _ Enter number of teeth lost to gum disease

66 All of them

88 Don't Know/Not Sure

99 Refused

**SECTION 3: Vascular Surgeries**

Q: Q3_1 ++++++++++++++++++++++++++++++++++++++++

I am now going to ask about surgeries or procedures you may have had on your heart or blood vessels.

Have you ever had coronary bypass surgery, such as a graft, CABG or a bypass procedure on the arteries of your heart?

1. Yes

2. No

8. Don't Know/Not Sure

9. Refused

IF (ANS = 1) SURG = 1

IF (ANS <> 1) SURG = 0

Q: Q3_2 ++++++++++++++++++++++++++++++++++++++++

Have you ever had a surgery or procedure on the arteries in your neck?

1. Yes

2. No

8. Don't Know/Not Sure

9. Refused

INTERVIEWER: If respondent seems unsure, add: This could include a carotid endarterectomy, which is surgery that opens and cleans the arteries on the side 4 of your neck, or carotid angioplasty possibly with stenting, which is a procedure where a wire with a balloon is threaded

from your leg through your body to the arteries in your neck and a balloon is used to open your artery and a coil may be placed to keep it open?

IF (ANS = 1) SURG = SURG + 1

Q: Q3_3 ++++++++++++++++++++++++++++++++++++++++

Have you ever had a repair of an aortic aneurysm?

1. Yes

2. No

8. Don't Know/Not Sure

9. Refused

IF (ANS = 1) SURG = SURG + 1

Q: Q3_4 ++++++++++++++++++++++++++++++++++++++++

Have you ever had a pacemaker implanted?

1. Yes

2. No

8. Don't Know/Not Sure

9. Refused

IF (ANS = 1) SURG = SURG + 1

Q: Q3_5 ++++++++++++++++++++++++++++++++++++++++

Have you ever had an angioplasty or stenting of a coronary artery with or without placing a coil in

the artery to keep it open?

1. Yes

2. No

8. Don't Know/Not Sure

9. Refused

IF (ANS = 1) SURG = SURG + 1

Q: Q3_6 ++++++++++++++++++++++++++++++++++++++++

Have you ever had a procedure to fix the arteries in your legs?

1. Yes

2. No

8. Don't Know/Not Sure

9. Refused

INTERVIEWER: If respondent seems unsure you can add: This could be a bypass or other surgery on the arteries of your leg including angioplasty of the leg arteries, which is a dilation of the arteries of the leg with a balloon.

IF (ANS = 1) SURG = SURG + 1

Q: Q3_6a ++++++++++++++++++++++++++++++++++++++++

Have you had a leg amputation?

1. Yes

2. No

8. Don't Know/Not Sure

9. Refused

IF (ANS = 1) SURG = SURG +1

Q: Q3_7 ++++++++++++++++++++++++++++++++++++++++

Have you ever had any other heart or blood vessel surgery?

1. Yes Specify:

2. No

8. Don't Know/Not Sure

9. Refused

IF (ANS = 1) SURG = SURG + 1

IF (SURG = 1) SKP Q3_8a

IF (SURG > 1) SKP Q3_8b

IF (SURG = 0) SKP Q4_1

Q: Q3_8a ++++++++++++++++++++++++++++++++++++++++

You just reported that you had a

IF (Q3_1 = 1) SHOW "coronary bypass surgery, a graft, CABG or bypass procedure."

IF (Q3_2 = 1) SHOW "surgery or procedure on the arteries in your neck."

IF (Q3_3 = 1) SHOW "repair of an aortic aneurysm."

IF (Q3_4 = 1) SHOW "pacemaker placed."

IF (Q3_5 = 1) SHOW "angioplasty or stenting of the coronary arteries."

IF (Q3_6 = 1) SHOW "procedure to fix the arteries in your legs."

IF (Q3_6a = 1) SHOW "leg amputation"

IF (Q3_7 = 1) SHOW Q3_7

How old were you when you had this done?

_ _ _ Enter age in years

If not sure, ask for best guess. If not possible to guess exact age use:

770 Less than 10 years old

771 Between 10 and 19

772 Between 20 and 29

773 Between 30 and 39

774 Between 40 and 49

775 Between 50 and 59

776 Between 60 and 69

777 Between 70 and 79

778 Between 80 and 89

779 Between 90 and 99

780 Over 99

888 Don't Know/Not Sure

999 Refused

SKP Q4_1

Q: Q3_8b ++++++++++++++++++++++++++++++++++++++++

You just reported that you had:

IF (Q3_1 = 1) SHOW "coronary bypass surgery, a graft, CABG or bypass procedure."

IF (Q3_2 = 1) SHOW "surgery or procedure on the arteries in your neck."

IF (Q3_3 = 1) SHOW "repair of an aortic aneurysm."

IF (Q3_4 = 1) SHOW "pacemaker placed."

IF (Q3_5 = 1) SHOW "angioplasty or stenting of the coronary arteries."

IF (Q3_6 = 1) SHOW "procedure to fix the arteries in your legs."

IF (Q3_6a = 1) SHOW "leg amputation"

IF (Q3_7 = 1) SHOW Q3_7

How old were you when you had the first of these procedures?

_ _ _ Enter age in years

If not sure, ask for best guess. If not possible to guess exact age use:

770 Less than 10 years old

771 Between 10 and 19

772 Between 20 and 29

773 Between 30 and 39

774 Between 40 and 49

775 Between 50 and 59

776 Between 60 and 69

777 Between 70 and 79

778 Between 80 and 89 779 Between 90 and 99

780 Over 99

888 Don't Know/Not Sure

999 Refused

Q: Q3_8c ++++++++++++++++++++++++++++++++++++++++

How old were you when you had the last of these procedures performed?

_ _ _ Enter age in years

If not sure, ask for best guess. If not possible to guess exact age use:

770 Less than 10 years old

771 Between 10 and 19

772 Between 20 and 29

773 Between 30 and 39

774 Between 40 and 49

775 Between 50 and 59

776 Between 60 and 69

777 Between 70 and 79

778 Between 80 and 89

779 Between 90 and 99

780 Over 99

888 Don't Know/Not Sure

999 Refused

IF (ANS > RESPAGE) AND IF (ANS < 769) REASK

**SECTION 4: Aspirin Use**

Q: Q4_1 ++++++++++++++++++++++++++++++++++++++++

Now I am going to ask you about your use of aspirin and other medications.

Do you take or have you ever taken aspirin or aspirin‑containing medicines for any reason?

Examples of aspirin‑containing medicine include: Bufferin, Anacin, Ascriptin or Ecotrin.

1. Yes

2. No

8. Don't Know/Not Sure

9. Refused

IF (ANS = 2) SKP Q4_3

IF (ANS = 1) SKP Q4_1b

IF (ANS = 8) SKP Q4_1a

IF (ANS = 9) SKP Q4_1a

Q: Q4_1a ++++++++++++++++++++++++++++++++++++++++

I am going to read you a list of compounds or medicines that contain aspirin. Please tell me if you now take or have ever taken any of them.

Aspirin Ascriptin Fiorinal

AlkaSltzer Pain Reliever Percodan Robaxisal

Goody's Extra Strength Gelpirin Damason‑P

Anacin Darvon Easprin

Excedrin Extra Strength Norgesic Empirin

Bayer Roxipran Azdone

BC Powder Soma Compound Supac

Bufferin Talwin Compound Zorprin

Ecotrin Orphengesic Equagesic

Cama Arthritis Pain Reliever

Do you now take or have you ever taken any of these?

1. Yes

2. No

8. Don't Know?Not Sure

9. Refused

IF (ANS > 1) SKP Q4_3

Q: Q4_1b +++++++++++++++++++++++++++++++++++++++++

Did a doctor or other health provider tell you to take this aspirin?

1. Yes

2. No

8. Don't Know/Not Sure

9. Refused

Q: Q4_2 ++++++++++++++++++++++++++++++++++++++++

At what age did you start taking aspirin or aspirin containing products regularly?

_ _ _ Enter age in years

If not sure, ask for best guess. If not possible to guess exact age use:

770 Less than 10 years old

771 Between 10 and 19

772 Between 20 and 29

773 Between 30 and 39

774 Between 40 and 49

775 Between 50 and 59

776 Between 60 and 69

777 Between 70 and 79

778 Between 80 and 89

779 Between 90 and 99

780 Over 99

887 Does not take aspirin regularly

888 Don't Know/Not Sure

999 Refused

IF (ANS > RESPAGE) AND IF (ANS < 769) REASK

Q: Q4_2a ++++++++++++++++++++++++++++++++++++++++

Are you currently taking aspirin or aspirin containing products regularly, that is, at least two times each week?

1. Yes

2. No

8. Don't Know/Not Sure

9. Refused

IF (ANS = 2) SKP Q4_2g

IF (ANS > 2) SKP Q4_3

Q: Q4_2b ++++++++++++++++++++++++++++++++++++++++

On average, how many days do you take aspirin or aspirin containing products each week?

__ Enter number of days

8 Don't Know/Not Sure

9 Refused

Q: Q4_2c ++++++++++++++++++++++++++++++++++++++++

How many aspirin do you take each day?

_ _ Enter number of aspirin

88 Don't Know/Not Sure

99 Refused

Q: Q4_2d ++++++++++++++++++++++++++++++++++++++++

What dosage do you normally take? Baby aspirin, half dose, normal dosage, or large or extra large?

1. Baby aspirin (approx 80 mg)

2. Half‑dose (approx 175 mg)

3. Normal (approx 325 mg)

4. Large or Extra (500 mg or more)

8. Don't Know/Not Sure

9. Refused

Q: Q4_2e ++++++++++++++++++++++++++++++++++++++++

For what purposes are you taking aspirin?

Is it to relieve pain?

1. Yes

2. No

8. Don't Know/Not Sure

9. Refused

Q: Q4_2f ++++++++++++++++++++++++++++++++++++++++

Is it to reduce the chance of a heart attack or stroke?

1. Yes

2. No

8. Don't Know/Not Sure

9. Refused

SKP Q4_3

Q: Q4_2g ++++++++++++++++++++++++++++++++++++++++

At what age did you STOP taking aspirin or aspirin containing products on a regular basis?

_ _ _ Enter age in years

If not sure, ask for best guess. If not possible to guess exact age use:

770 Less than 10 years old

771 Between 10 and 19

772 Between 20 and 29

773 Between 30 and 39

774 Between 40 and 49

775 Between 50 and 59

776 Between 60 and 69

777 Between 70 and 79

778 Between 80 and 89

779 Between 90 and 99

780 Over 99

888 Don't Know/Not Sure

999 Refused

IF (ANS > RESPAGE) AND IF (ANS < 769) REASK

Q: Q4_3 ++++++++++++++++++++++++++++++++++++++++

Have you ever taken Nonsteroidal Anti‑Inflammatory agents on a regular basis, that is at least two times each week?

Examples of Nonsteriodal Anti‑Inflammatory agents include: Advil, Motrin, Ibuprofen and Naprosyn.

1. Yes

2. No

8. Don't Know/Not Sure

9. Refused

IF (ANS = 1) SKP Q4_4

IF (ANS = 2) SKP Q5_1

IF (ANS = 8) SKP Q4_3a

IF (ANS = 9) SKP Q4_3a

Q: Q4_3a ++++++++++++++++++++++++++++++++++++++++

I am now going to read you a list of medicines that are considered Non Steroidal Anti Inflammatory agents. Please tell me if you now take or have ever taken any of them.

Aleve Disalcid Dilobid or Diflunisal

Naprosyn Etodalac Feledene or Piroxicam

Ibuprofen Disalcid Tolectin or Tolmetin

Lodine Mono Gesic Votaren or Diclofenac

Advil Salflex Daypro or Oxyprozin

Motrin Sulindac Actron or Ketoprofen

Relafen Toradol Cataflam or Diclofenac

Nuprin Trilisate Nebumetone

Orudis Fenoprofen IAU Tablets

Clinoril Indocin Metenamic Acid

Celebrex

Do you now take or have you ever taken any of these?

1. Yes

2. No

8. Don't Know/Not Sure

9. Refused

IF (ANS > 1) SKP Q5_1

Q: Q4_4 ++++++++++++++++++++++++++++++++++++++++

Are you currently taking Non Steroidal Anti Inflammatory medications regularly, that is at least twice a week?

1. Yes

2. No

8. Don't Know/Not Sure

9. Refused

**SECTION 5: Reproductive History**

IF (GENDER = "M") SKP Q6_1

Q: Q5_1 ++++++++++++++++++++++++++++++++++++++++

Now I am going to ask you some questions related to women's health.

How many times have you been pregnant?

_ _ _ Number of pregnancies

777 Never been pregnant

888 Don't Know/Not Sure

999 Refused

IF (ANS > 776) SKP Q5_2

Q: Q5_1a ++++++++++++++++++++++++++++++++++++++++

How many live births have you had?

_ _ _ Number of live births

777 No live births

888 Don't Know/Not Sure

999 Refused

Q: Q5_2 ++++++++++++++++++++++++++++++++++++++++

Have you gone through menopause or the change of life?

1. Yes

2. No

8. Don't Know/Not Sure

9. Refused

IF (ANS = 2) SKP Q5_3

IF (ANS = 9) SKP Q5_3

Q: Q5_2a ++++++++++++++++++++++++++++++++++++++++

How old were you at the time of your last natural menstrual period?

_ _ _ Age in years

888 Don't Know/Not Sure

999 Refused

IF (ANS > RESPAGE)AND IF (ANS < 888) REASK

Q: Q5_3 ++++++++++++++++++++++++++++++++++++++++

Have you ever had a hysterectomy, that is, surgery to remove your uterus or womb?

1. Yes

2. No

8. Don't Know/Not Sure

9. Refused

IF (ANS <> 1) SKP Q5_5

Q: Q5_3a ++++++++++++++++++++++++++++++++++++++++

At what age?

_ _ _ Enter age

888 Don't Know/Not Sure

999 Refused

IF (ANS > RESPAGE) AND IF (ANS < 888) REASK

Q: Q5_5 ++++++++++++++++++++++++++++++++++++++++

Have you ever had an ovary removed?

1. Yes

2. No

8. Don't Know/Not Sure

9. Refused

IF (ANS <> 1) SKP Q5_6

Q: Q5_5a ++++++++++++++++++++++++++++++++++++++++

At what age?

_ _ _ Enter age

888 Don't Know/Not Sure

999 Refused

INTERVIEWER: If respondent has had ovaries removed at two different times, record age when the last one was removed.

IF (ANS > RESPAGE) AND IF (ANS < 888) REASK

Q: Q5_5b ++++++++++++++++++++++++++++++++++++++++

How many ovaries were removed?

1. One

2. Both

8. Don't Know

9. Refused

Q: Q5_6 ++++++++++++++++++++++++++++++++++++++++

Have you had any other condition or treatment that caused your menstrual periods to permanently

stop, other than the surgeries we just discussed or natural menopause?

1. Yes Specify:

2. No

8. Don't Know/Not Sure

9. Refused

IF (ANS > 1) SKP Q5_7

Q: Q5_6a ++++++++++++++++++++++++++++++++++++++++

How old were you when this occurred?

_ _ _ Enter age

888 Don't Know/Not Sure

999 Refused

IF (ANS > RESPAGE) AND IF (ANS < 888) REASK

Q: Q5_7 ++++++++++++++++++++++++++++++++++++++++

Have you ever taken oral contraceptives, birth control pills, or used Depo Provera or Norplant to

prevent pregnancy, but not for hormone replacement?

1. Yes

2. No

8. Don't Know/Not Sure

9. Refused

IF (ANS > 1) SKP Q5_8

Q: Q5_7b ++++++++++++++++++++++++++++++++++++++++

Keeping in mind that you may have started and stopped several times, please estimate the total number of years that you took birth control pills, Depo Provera or Norplant.

_ _ _ Enter number of years

888 Don't Know/Not Sure

999 Refused

Q: Q5_8 ++++++++++++++++++++++++++++++++++++++++

Women sometimes take female hormones. They are taken for a variety of reasons including hot flashes or other symptoms, of menopause and sometimes for the prevention of bone loss. Have you ever taken hormone or estorgen replacement therapy such as hormone pills or shots or used hormone patches such as Premarin, Estrace, Ogen or any other estrogens?

1. Yes

2. No

8. Don't Know/Not Sure

9. Refused

IF (ANS > 1) SKP Q6_1

Q: Q5_8a ++++++++++++++++++++++++++++++++++++++++

Are you currently on these medications?

1. Yes

2. No

8. Don't Know/Not Sure

9. Refused

IF (ANS = 2) SKP Q5_8d

IF (ANS = 8) SKP Q6_1

IF (ANS = 9) SKP Q6_1

Q: Q5_8b ++++++++++++++++++++++++++++++++++++++++

How old were you when you started taking these hormones?

_ _ _ Enter age in years

If not sure, ask for best guess. If not possible to guess exact age use:

770 Less than 10 years old

771 Between 10 and 19

772 Between 20 and 29

773 Between 30 and 39

774 Between 40 and 49

775 Between 50 and 59

776 Between 60 and 69

777 Between 70 and 79

778 Between 80 and 89

779 Between 90 and 99

780 Over 99

888 Don't Know/Not Sure

999 Refused

IF (ANS > RESPAGE) AND IF (ANS < 769) REASK

Q: Q5_8c ++++++++++++++++++++++++++++++++++++++++

Do you have menstrual periods regularly while taking these hormones?

1. Yes

2. No

8. Don't Know/Not Sure

9. Refused

IF (Q5_3 = 1) SKP Q6_1

SKP Q6_1

Q: Q5_8d ++++++++++++++++++++++++++++++++++++++++

How old were you when you started taking these hormones?

_ _ _ Enter age in years

If not sure, ask for best guess. If not possible to guess exact age use:

770 Less than 10 years old

771 Between 10 and 19

772 Between 20 and 29

773 Between 30 and 39

774 Between 40 and 49

775 Between 50 and 59

776 Between 60 and 69

777 Between 70 and 79

778 Between 80 and 89

779 Between 90 and 99

780 Over 99

888 Don't Know/Not Sure

999 Refused

IF (ANS > RESPAGE) AND IF (ANS < 769) REASK

Q: Q5_8e ++++++++++++++++++++++++++++++++++++++++

How old were you when you stopped taking these hormones?

_ _ _ Enter age in years

If not sure, ask for best guess. If not possible to guess exact age use:

770 Less than 10 years old

771 Between 10 and 19

772 Between 20 and 29

773 Between 30 and 39

774 Between 40 and 49

775 Between 50 and 59

776 Between 60 and 69

777 Between 70 and 79

778 Between 80 and 89

779 Between 90 and 99

780 Over 99

888 Don't Know/Not Sure

999 Refused

IF (ANS > RESPAGE) AND IF (ANS < 769) REASK

Q: Q5_8f ++++++++++++++++++++++++++++++++++++++++

Did you have monthly menstrual periods while you were taking these hormones?

1. Yes

2. No

8. Don't Know/Not Sure

9. Refused

**SECTION 6: Smoking**

Q: Q6_1 ++++++++++++++++++++++++++++++++++++++++

Now I am going to ask about your smoking habits.

Have you smoked at least 100 cigarettes in your lifetime?

1. Yes

2. No

8. Don't Know/Not Sure

9. Refused

IF (ANS > 1) SKP Q6_11

Q: Q6_1a ++++++++++++++++++++++++++++++++++++++++

Do you smoke cigarettes now, even occasionally?

1. Yes

2. No

8. Don't Know/Not Sure

9. Refused

IF (ANS > 1) SKP Q6_7

Q: Q6_2 ++++++++++++++++++++++++++++++++++++++++

How old were you when you started smoking?

_ _ _ Enter age in years

888 Don't Know/Not Sure

999 Refused

IF (ANS > RESPAGE) AND IF (ANS < 888) REASK

Q: Q6_3 ++++++++++++++++++++++++++++++++++++++++

For how many years have you been a smoker? Do not include times you may have stopped smoking.

_ _ _ Enter number of years

888 Don't Know/Not Sure

999 Refused

IF (ANS > RESPAGE) AND IF (ANS < 888) REASK

Q: Q6_4 ++++++++++++++++++++++++++++++++++++++++

On average, over this period, how many cigarettes did you smoke per day, per week or per month?

_ _ _ Enter number of cigarettes 1 pack = 20 cigarettes

101 ‑ 199 Number per day

201 ‑ 299 Number per week

301 ‑ 399 Number per month

401 ‑ 499 Number per year

888 Don't Know/Not Sure

999 Refused

Q: Q6_5 ++++++++++++++++++++++++++++++++++++++++

How many cigarettes do you currently smoke?

_ _ _ Enter number of cigarettes 1 pack = 20 cigarettes

101 ‑ 199 Number per day

201 ‑ 299 Number per week

301 ‑ 399 Number per month

401 ‑ 499 Number per year

888 Don't Know/Not Sure

999 Refused

SKP Q6_13

Q: Q6_7 ++++++++++++++++++++++++++++++++++++++++

How old were you when you started smoking?

_ _ _ Enter age in years

888 Don't Know/Not Sure

999 Refused

Q: Q6_8 ++++++++++++++++++++++++++++++++++++++++

How old were you when you stopped smoking?

_ _ _ Enter age in years

888 Don't Know/Not Sure

999 Refused

IF (ANS > RESPAGE) AND IF (ANS < 888) REASK

Q: Q6_9 ++++++++++++++++++++++++++++++++++++++++

For how many years were you a smoker? Do not include times you may have stopped smoking?

_ _ _ Enter number of years

888 Don't Know/Not Sure

999 Refused

IF (ANS > RESPAGE) AND IF (ANS < 888) REASK

Q: Q6_10 ++++++++++++++++++++++++++++++++++++++++

During this period, how many cigarettes did you smoke on average per day, per week or per month?

_ _ _ Enter number of cigarettes 1 pack = 20 cigarettes

101 ‑ 199 Number per day

201 ‑ 299 Number per week

301 ‑ 399 Number per month

401 ‑ 499 Number per year

888 Don't Know/Not Sure

999 Refused

Q: Q6_11 ++++++++++++++++++++++++++++++++++++++++

Does anyone living with you smoke cigarettes regularly?

1. Yes

2. No

8. Don't Know/Not Sure

9. Refused

IF (Q6_1a = 1) SKP Q6_13

Q: Q6_12 ++++++++++++++++++++++++++++++++++++++++

During the past year, about how many hours PER WEEK, on the average, were you in close contact with people when they were smoking? For example, in your home, in a car, at work or other close quarters.

_ _ _ Enter Number of Hours

777 None

888 Don't Know/Not Sure

999 Refused

Q: Q6_13 ++++++++++++++++++++++++++++++++++++++++

Do you currently smoke cigars, cigarillos or a pipe, even occasionally?

1. Yes

2. No

8. Don't Know/Not Sure

9. Refused

Q: Q6_14a ++++++++++++++++++++++++++++++++++++++++

Have you ever used or tried any smokeless tobacco products such as chewing tobacco or snuff?

1. Yes

2. No

8. Don't Know/Not Sure

9. Refused

IF (ANS > 1) SKP Q7_1

Q: Q6_14b ++++++++++++++++++++++++++++++++++++++++

Do you currently use chewing tobacco or snuff every day, some days, or not at all?

1. Every day

2. Some days

3. Not at all

8. Don't Know/Not Sure

9. Refused

**SECTION 7: Alcohol Use**

Q: Q7_1 ++++++++++++++++++++++++++++++++++++++++

I am now going to ask about your use of alcoholic beverages.

Do you presently drink alcoholic beverages, including beer, wine, and other drinks made with hard liquor, even occasionally?

1. Yes

2. No

8. Don't Know/Not Sure

9. Refused

IF (ANS > 1) SKP Q7_6

Q: Q7_2 ++++++++++++++++++++++++++++++++++++++++

How old were you when you first started drinking alcoholic beverages regularly? By regularly, we mean at least 1 drink per month for 1 year.

_ _ _ Enter age in years

777 Does not drink regulary

888 Don't Know/Not Sure

999 Refused

IF (ANS > RESPAGE) AND IF (ANS < 777) REASK

IF (ANS = 777) SKP Q7_6

IF (ANS = 888) SKP Q7_4

IF (ANS = 999) SKP Q7_4

Q: Q7_3 ++++++++++++++++++++++++++++++++++++++++

Did you ever stop drinking regularly for more than one year and then start drinking regularly again?

1. Yes

2. No

7. Has never drunk regularly

8. Don't Know/Not Sure

9. Refused

If (ANS = 2) SKP Q7_4

IF (ANS = 7) SKP Q7_6

IF (ANS > 7) SKP Q7_4

Q: Q7_3a ++++++++++++++++++++++++++++++++++++++++

For how many years did you not drink regularly?

_ _ _ Enter number of years

888 Don' Know/Not Sure

999 Refused

INTERVIEWER: Probe number of years participant did not drink regularly or drank less than

1 drink per month

Q: Q7_4 ++++++++++++++++++++++++++++++++++++++++

Thinking over the years that you used alcoholic beverages, on average, about how many drinks did you usually have? For example, one per day, three per week, and so on. Please include beer, wine and hard liquor.

_ _ _ Enter number of drinks

101‑125 Drinks per day

201‑299 Drinks per week

301‑399 Drinks per month

401‑499 Drinks per year

888 Don't Know/Not Sure

999 Refused

Q: Q7_5 ++++++++++++++++++++++++++++++++++++++++

How many alcoholic beverages do you presently drink?

_ _ _ Enter number of drinks

101‑125 Drinks per day

201‑299 Drinks per week

301‑399 Drinks per month

401‑499 Drinks per year

888 Don't Know/Not Sure

999 Refused

SKP Q8_1

Q: Q7_6 ++++++++++++++++++++++++++++++++++++++++

Have you ever drank alcoholic beverages, including beer, wine, and other drinks made with hard liquor on a regular basis? By regular, we mean at least 1 drink per month for 1 year.

1. Yes

2. No

8. Don't Know/Not Sure

9. Refused

IF (ANS > 1) SKP Q8_1

Q: Q7_7 ++++++++++++++++++++++++++++++++++++++++

How old were you when you first started drinking alcoholic beverages regularly? By regularly, we mean at least 1 drink per month for 1 year.

_ _ _ Enter age in years

888 Don't Know/Not Sure

999 Refused

IF (ANS > RESPAGE) AND IF (ANS < 888) REASK

Q: Q7_8 ++++++++++++++++++++++++++++++++++++++++

For how many years did you regularly drink alcoholic beverages? Do not include times you may have stopped drinking regularly.

_ _ _ Enter number of years

888 Don't Know/Not Sure

999 Refused

IF (ANS > RESPAGE) AND IF (ANS < 888) REASK

**SECTION 8: Physical Activity**

Q: Q8_1 ++++++++++++++++++++++++++++++++++++++++

I am now going to ask about how much exercise you do.

How many times per week do you engage in intense physical activity, enough to work up a sweat?

_ _ _ Enter times per week

777 None

888 Don't Know/Not Sure

999 Refused

Q: Q8_2 ++++++++++++++++++++++++++++++++++++++++

How would you compare your activity level to others your age? Would you say you are less

active, about the same or more active?

1. Less active

2. Same as others your age

3. More active

8. Don't Know/Not Sure

9. Refused

Q: Q8_3 ++++++++++++++++++++++++++++++++++++++++

During the last year, have you had a fall? Do not include falls during skiing, skating or other activities that may affect balance.

1. Yes

2. No

8. Don't Know/Not Sure

9. Refused

IF (ANS > 1) SKP Q9_1

Q: Q8_3a ++++++++++++++++++++++++++++++++++++++++

How many times have you fallen in the last year?

_ _ _ Enter number of falls

888 Don't Know/Not Sure

999 Refused

**SECTION 9: General Health**

Q: Q9_1 ++++++++++++++++++++++++++++++++++++++++

In the next set of questions, I will ask about your general health.

In general, would you say that your health is excellent, very good, good, fair or poor?

1. Excellent

2. Very good

3. Good

4. Fair

5. Poor

8. Don't Know/Not Sure

9. Refused

Q: Q9_2 ++++++++++++++++++++++++++++++++++++++++

Now I'm going to read a list of activities that you might do during a typical day. As I read each item, please tell me if your health now limits you a lot, limits you a little or does not limit you at all in these activities.

Moderate activities such as moving a table, pushing a vacuum cleaner, bowling or playing golf. Does your health now limit you a lot, a little or not at all in these activities?

1. Yes, limited a lot

2. Yes, limited a little

3. No, not limited at all

8. Don't Know/Not Sure

9. Refused

INTERVIEWER: If respondent indicates that he/she does not do any of these activities probe with: "Is that because of your health?" If the answer is "yes" code either 1 or 2. If the answer is "no" code 3

Q: Q9_3 ++++++++++++++++++++++++++++++++++++++++

Climbing several flights of stairs? Does your health now limit you a lot, limit you a little or not limit you at all in climbing several flights of stairs?

1. Yes, limited a lot

2. Yes, limited a little

3. No, not limited at all

8. Don't Know/Not Sure

9. Refused

INTERVIEWER: If respondent indicates that he/she does not do any of these activities probe with: "Is that because of your health?" If the answer is "yes" code either 1 or 2. If the answer is "no" code 3

Q: Q9_4 ++++++++++++++++++++++++++++++++++++++++

The following two questions ask about your physical health and daily activities.

During the past 4 weeks, have you accomplished less than you would like as a result of your physical health?

1. Yes

2. No

8. Don't Know/Not Sure

9. Refused

Q: Q9_5 ++++++++++++++++++++++++++++++++++++++++

During the past 4 weeks, were you limited in the kind of work or other regular daily activities you do as a result of your physical health?

1. Yes

2. No

8. Don't Know/Not Sure

9. Refused

Q: Q9_6 ++++++++++++++++++++++++++++++++++++++++

The following two questions ask about your emotions and your daily activities.

During the past 4 weeks, have you accomplished less than you would like as a result of any emotional problems, such as feeling depressed or anxious?

1. Yes

2. No

8. Don't Know/Not Sure

9. Refused

Q: Q9_7 ++++++++++++++++++++++++++++++++++++++++

During the past 4 weeks, did you not do work or other regular daily activities as carefully as usual as a result of any emotional problems, such as feeling depressed or anxious?

1. Yes

2. No

8. Don't Know/Not Sure

9. Refused

Q: Q9_8 ++++++++++++++++++++++++++++++++++++++++

During the past 4 weeks, how much did pain interfere with your normal work, including both work outside the home and housework? Did it interfere not at all, a little bit, moderately, quite a bit or extremely?

1. Not at all

2. A little bit

3. Moderately

4. Quite a bit

5. Extremely

8. Don't Know/Not Sure

9. Refused

Q: Q9_9 ++++++++++++++++++++++++++++++++++++++++

During the past 4 weeks, how much of the time has your physical health or emotional problems interfered with your social activities like visiting with friends or relatives? Has it interfered all of the time, most of the time, some of the time, a little of the time or none of the time?

1. All of the time

2. Most of the time

3. Some of the time

4. A little of the time

5. None of the time

8. Don't Know/Not Sure

9. Refused

Q: Q9_10 ++++++++++++++++++++++++++++++++++++++++

How much of the time during the past 4 weeks have you felt calm and peaceful? Would you say all of the time, most of the time, a good bit of the time, some of the time, a little of the time or

none of the time?

1. All of the time

2. Most of the time

3. A good bit of the time

4. Some of the time

5. A little of the time

6. None of the time

8. Don't Know/Not Sure

9. Refused

Q: Q9_11 ++++++++++++++++++++++++++++++++++++++++

How much of the time during the past 4 weeks did you have a lot of energy? Would you say all of the time, most of the time, a good bit of the time, some of the time, a little of the time or none of the time?

1. All of the time

2. Most of the time

3. A good bit of the time

4. Some of the time

5. A little of the time

6. None of the time

8. Don't Know/Not Sure

9. Refused

Q: Q9_12 ++++++++++++++++++++++++++++++++++++++++

How much of the time during the past 4 weeks did you feel downhearted and blue? Would you say all of the time, most of the time, a good bit of the time, some of the time, a little of the time or none of the time?

1. All of the time

2. Most of the time

3. A good bit of the time

4. Some of the time

5. A little of the time

6. None of the time

8. Don't Know/Not Sure

9. Refused

**SECTION 10: Social Network**

Q: Q10_1 ++++++++++++++++++++++++++++++++++++++++

Next, I'm going to ask just a couple of questions about the friends and relatives you feel close to.

How many close friends do you have? That is, people that you feel at ease with, can talk to about private matters, and can call on for help?

_ _ _ Enter number

777 None

888 Don't Know/Not Sure

999 Refused

IF (ANS > 50) AND IF (ANS < 776) REASK

Q: Q10_2 ++++++++++++++++++++++++++++++++++++++++

How many relatives do you have that you feel close to?

_ _ _ Enter number

777 None

888 Don't Know/Not Sure

999 Refused

IF (ANS > 50) AND IF (ANS < 776) REASK

IF (Q10_1 >= 777) AND IF (Q10_2 >= 777) SKP Q11_1

Q: Q10_3 ++++++++++++++++++++++++++++++++++++++++

How many of these friends or relatives do you see at least once a month?

_ _ _ Enter number

777 None

888 Don't Know/Not Sure

999 Refused

**SECTION 11: Depression and Cohen’s Perceived Stress Scale**

Q: Q11_1 ++++++++++++++++++++++++++++++++++++++++

These next questions are about how you might have felt or behaved recently. Please tell me how often you felt this way during the past week.

During the past week, would you say you felt depressed less than one day, one to two days, three to four days or five to seven days?

1 less than 1 day

2 1‑2 days

3 3‑4 days

4 5‑7 days

8 Don't Know/Not Sure

9 Refused

Q: Q11_2 ++++++++++++++++++++++++++++++++++++++++

During the past week, would you say that you felt lonely less than one day, one to two days, three to four days, or five to seven days?

1 less than 1 day

2 1‑2 days

3 3‑4 days

4 5‑7 days

8 Don't Know/Not Sure

9 Refused

Q: Q11_3 ++++++++++++++++++++++++++++++++++++++++

During the past week would you say that you had crying spells less than one day, one to two days, three to four days, or five to seven days?

1 less than 1 day

2 1‑2 days

3 3‑4 days

4 5‑7 days

8 Don't Know/Not Sure

9 Refused

Q: Q11_4 ++++++++++++++++++++++++++++++++++++++++

During the past week, would you say that you felt sad less than one day, one to two days, three to four days, or five to seven days?

1 less than 1 day

2 1‑2 days

3 3‑4 days

4 5‑7 days

8 Don't Know/Not Sure

9 Refused

Q: Q11_5 ++++++++++++++++++++++++++++++++++++++++

In the last month, how often have you felt that you were unable to control the important things in your life? Would you say Never, Almost never, Sometimes, Fairly often, or Very often?

1. Never

2. Almost never

3. Sometimes

4. Fairly often

5. Very often

8. Don't Know/Not Sure

9. Refused

Q: Q11_6 ++++++++++++++++++++++++++++++++++++++++

In the last month, how often have you felt confident about your ability to handle your personal problems? Would you say Never, Almost never, Sometimes, Fairly often, or Very often?

1. Never

2. Almost never

3. Sometimes

4. Fairly often

5. Very often

8. Don't Know/Not Sure

9. Refused

Item Q11_6a added 8/17/05 to correct the perceived stress scale per LeaVonne Pulley.

Q: Q11_6a ++++++++++++++++++++++++++++++++++++++++++

In the last month, how often have you felt that things were going your way?

Woud you say never, almost never, sometimes, fairly often or very often?

1. Never

2. Almost never

3. Sometimes

4. Fairly often

5. Very often

8. Don't Know/Not Sure

9. Refused

Q: Q11_7 ++++++++++++++++++++++++++++++++++++++++

In the last month, how often have you found that you could not cope with all the things that you had to do? Would you say never, almost never, sometimes, fairly often or very often?

1. Never

2. Almost never

3. Sometimes

4. Fairly often

5. Very often

8. Don't Know/Not Sure

9. Refused

Q: Q11_8 ++++++++++++++++++++++++++++++++++++++++

In the last month, how often have you felt difficulties were piling up so high that you could not overcome them? Would you say never, almost never, sometimes, fairly often or very often?

1. Never

2. Almost never

3. Sometimes

4. Fairly often

5. Very often

8. Don't Know/Not Sure

9. Refused

Time urgency/achievement striving and work status added 7/29/03

I am going to list for you some traits or qualities that describe people. For each one, please tell me whether that trait describes you very well, fairly well, somewhat or not at all.

11.8.1 Would you say that the strong need to excel or be best in most things describes you very well, fairly well, somewhat or not at all?

1. Very well

2. Fairly well

3. Somewhat

4. Not at all

8. Don't Know/Not Sure

9. Refused

11.8.2 The need to be bossy or dominating. Does that describe you very well, fairly well, somewhat or not at all?

1. Very well

2. Fairly well

3. Somewhat

4. Not at all

8. Don't Know/Not Sure

9. Refused

11.8.3 Usually feeling pressured for time. Does that describe you very well, fairly well, somehwat or not at all?

1. Very well

2. Fairly well

3. Somewhat

4. Not at all

8. Don't Know/Not Sure

9. Refused

11.8.4 Being hard driving and competitive. Does that describe you very well, fairly well, somewhat or not at all?

1. Very well

2. Fairly well

3. Somewhat

4. Not at all

8. Don't Know/Not Sure

9. Refused

11.8.5 Eating too quickly. Does that describe you very well, fairly well, somewhat or not at all?

1. Very well

2. Fairly well

3. Somewhat

4. Not at all

8. Don't Know/Not Sure

9. Refused

11.8.6 Do you get quite upset when you have to wait for anything?

1. Yes

2. No

8. Don't Know/Not Sure

9. Refused

11.8.7 Are you currently employed for wages, self-employed, out of work for more than 1 year, out of work for less than 1 year, a homemaker, a student, retired, or unable to work?

1. Employed for wages Skip to Q11.8.8

2. Self-employed Skip to Q11.8.8

3. Out of work for more than 1 year Skip to Q11.8.16

4. Out of work for less than 1 year Skip to Q11.8.16

5. Homemaker Skip to Q11.8.12

6. Student Skip to Q11.8.8

7. Retired Skip to Q11.8.16

8. Unable to work Skip to Q11.8.16

9. Refused

For those employed for wages, self-employed or a student

11.8.8 Have you often felt very pressed for time?

1. Yes

2. No

8. Don't Know/Not Sure

9. Refused

11.8.9 Has you work often stayed with you so that you were thinking about it after working hours, or all day long?

1. Yes

2. No

8. Don't Know/Not Sure

9. Refused

11.8.10 Has you work or daily work activity often stretched you to very limits of your energy and capacity?

1. Yes

2. No

8. Don't Know/Not Sure

9. Refused

11.8.11 Have you often felt uncertain, uncomfortable or dissatisfied with how well you were doing in your regular line of work or daily work activity?

1. Yes Skip to Q11.8.20

2. No Skip to Q11.8.20

8. Don't Know/Not Sure Skip to Q11.8.20

9. Refused Skip to Q11.8.20

For homemakers only

11.8.12 Have you often felt very pressed for time?

1. Yes

2. No

8. Don't Know/Not Sure

9. Refused

11.8.13 Have you often had a feeling of dissatisfaction?

1. Yes

2. No

8. Don't Know/Not Sure

9. Refused

11.8.14 Has your housework often stayed with you so that you think about it all the time?

1. Yes

2. No

8. Don't Know/Not Sure

9. Refused

11.8.15 In general, do you find housework a big strain?

1. Yes Skip to Q11.8.20

2. No Skip to Q11.8.20

8. Don't Know/Not Sure Skip to Q11.8.20

9. Refused Skip to Q11.8.20

For unemployed, retired or disabled

11.8.16 Have you often felt very pressed for time?

1. Yes

2. No

8. Don't Know/Not Sure

9. Refused

11.8.17 Have things you needed to do stayed with you so that you were thinking about them all day long?

1. Yes

2. No

8. Don't Know/Not Sure

9. Refused

11.8.18 Have you often felt stretched to the very limits of your capacity and energy?

1. Yes

2. No

8. Don't Know/Not Sure

9. Refused

11.8.19 Have you often felt uncertain, uncomfortable or dissatisfied with your usual activities?

1. Yes

2. No

8. Don't Know/Not Sure

9. Refused

11.8.20 How satisfied are you with your life as a whole? Would you say you are very satisfied, somewhat satisfied, neither satisfied nor dissatisfied, somehwat dissatisfied or very dissatisfied?

1. Very satisfied

2. Somewhat satisfied

3. Neither satisfied nor dissatisfied

4. Somewhat dissatisfied

5. Very dissatisfied

8. Don't Know/Not Sure

9. Refused

11.8.21 Up to now, have you been able to satisfy most of your ambitions in life or have you had to settle for less than you hoped for?

1. Satisfied most ambitions

2. Have had to settle for less

8. Don't Know/Not Sure

9. Refused

11.8.22 Do you think you have had a fair opportunity to make the most of yourself in life, or have you been held back in some ways?

1. Have had a fair opportunity

2. Have been held back

8. Don't Know/Not Sure

9. Refused

Six item cognitive screener added on 12/18/03

Q: Q11_9

Now I would like to ask you some questions that ask you to use your memory.

11.9 I am going to name three objects. Please wait until I say all three words, then repeat them. Remember what they are because I am going to ask you to name them again in a few minutes, but please do not write anything down. Please repeat these words for me:

apple, table, penny.

INTERVIEWER: Did respondent correctly repeat all three words?

1. Yes

2. No

INTERVIEWER: You may repeat the three words, apple, table and penny up to three times if necessary.

CogScore = 0

Q: Q11_10

11.10 Now, without looking at a calendar or watch, what year is this?

INTERVIEWER: Current year = CATI SHOWS CURRENT DAY

1. Respondent answered correctly

2. Respondent answered incorrectly

IF (ANS = 1) CogScore = CogScore + 1

IF (ANS = 2) CogScore = CogScore + 0

Q: Q11_11

11.11 Without looking at a calendar or watch, what month is this?

INTERVIEWER: Current month = CATI SHOW CURRENT MONTH

1. Respondent answered correctly

2. Respondent answered incorrectly

IF (ANS = 1) CogScore = CogScore + 1

IF (ANS = 2) CogScore = CogScore + 0

Q: Q11_12

11.12 Without looking at a calendar or watch, what is the day of the week?

INTERVIEWER: Today is CATI SHOW CURRENT DAY

1. Respondent answered correctly

2. Respondent answered incorrectly

IF (ANS = 1) CogScore = CogScore + 1

IF (ANS = 2) CogScore = CogScore + 0

Q: Q11_13

11.13 What were the three objects I asked you to remember?

Items = Apple, Table, Penny Any order is acceptable

1. Respondent able to remember 1 item

2. Respondent able to remember 2 items

3. Respondent able to remember all 3 items

4. Respondent unable to remember any of the items

INTERVIEWER: Any order is acceptable. Do not prompt

If respondent remembers 0 items CogScore = CogScore + 0

If respondent remembers 1 item CogScore = CogScore +1

If respondent remembers 2 items CogScore = CogScore + 2

If respondent remembers 3 items CogScore = CogScore + 3

**SECTION 12: Demographics II**

Q: Q12_1 ++++++++++++++++++++++++++++++++++++++++

What is your marital status? Are you married, divorced, widowed, separated or have you never been married?

1 Married

2 Divorced

3 Widowed

4 Separated

5 Never been married

8 Don't Know/Not Sure

9 Refused

IF (ANS = 1) SKP Q12_3

Q: Q12_2 ++++++++++++++++++++++++++++++++++++++++

Are you currently living with someone in a marriage‑like relationship?

1. Yes

2. No

8. Don't Know/Not Sure

9. Refused

Q: Q12_3 ++++++++++++++++++++++++++++++++++++++++

Do you have any kind of healthcare coverage such as health insurance, an HMO or a government plan like Medicare or Medicaid?

1. Yes

2. No

8. Don't Know/Not Sure

9. Refused

Q: Q12_4 ++++++++++++++++++++++++++++++++++++++++

What is the highest grade or year of school you have completed?

(READ ONLY IF NECESSARY)

11 Never attended or kindergarten only

12 Eighth grade or less

13 Some high school (9th ‑ 11th grade)

14 High school graduate or GED certificate

15 Some technical school

16 Technical school graduate

17 Some college

18 College graduate

19 Postgraduate or professional degree

88 Don't Know/Not Sure

99 Refused

IF (Q12_1 > 1) AND IF (Q12_2 > 1) SKP Q12_5

Q: Q12_4a ++++++++++++++++++++++++++++++++++++++++

What is the highest grade or year of school your spouse or partner has completed?

(READ ONLY IF NECESSARY)

11 Never attended or kindergarten only

12 Eighth grade or less

13 Some high school (9th ‑ 11th grade)

14 High school graduate or GED certificate

15 Some technical school

16 Technical school graduate

17 Some college

18 College graduate

19 Postgraduate or professional degree

88 Don't Know/Not Sure

99 Refused

Q: Q12_5 ++++++++++++++++++++++++++++++++++++++++

Not counting yourself, how many adults, age 18 or older currently live in the same household with you?

_ _ Enter number of adults

77 No other adults live in home

88 Don't Know/Not Sure

99 Refused

INTERVIEWER: Please note, this is asking for additional adults, so if respondent says "just 1" verify whether he/she means one other person lives there or that he/she is the only adult resident.

IF (ANS > 12) AND IF (ANS < 76) REASK

Q: Q12_6 ++++++++++++++++++++++++++++++++++++++++

How many children, age 17 or younger, currently live in the same household with you?

_ _ Enter number of children

77 No children in home

88 Don't Know/Not Sure

99 Refused

IF (ANS > 12) AND IF (ANS < 76) REASK

Q: Q12_7 ++++++++++++++++++++++++++++++++++++++++

If you had a serious illness or became disabled, do you have someone who would be able to provide care for you on an on‑going basis?

1. Yes

2. No

8. Don't Know/Not Sure

9. Refused

IF (ANS > 1) SKP Q12_11

Q: Q12_8 ++++++++++++++++++++++++++++++++++++++++

Who would that most likely be?

INTERVIEWER: Get first and last name

__________________ ____________________

Q: Q12_9 ++++++++++++++++++++++++++++++++++++++++

What relationship is that person to you? For example, is it your spouse, partner or significant other a daughter, a son, daughter‑in‑law, son‑in‑law, sister, brother, granddaughter, grandson, niece, nephew, other relative, friend or neighbor?

11. Spouse

12. Partner, significant other, boyfriend or girlfriend

13. Daughter

14. Son

15. Daughter in law

16. Son in law

17. Sister

18. Brother

19. Granddaughter

20. Grandson

21. Niece

22. Nephew

23. Other Relative Specify:

24. Friend or neighbor

88. Don't Know/Not Sure

99 Refused

Q: Q12_10 ++++++++++++++++++++++++++++++++++++++++

Does <FILL NAME FROM Q12_8> currently live with you?

1. Yes

2. No

8. Don't Know/Not Sure

9. Refused

Q: Q12_11 ++++++++++++++++++++++++++++++++++++++++

Are you currently providing care on an on‑going basis to a family member with a chronic illness

or disability? This includes any kind of help such as watching your family member, dressing or bathing this person, arranging care, or providing transportation?

1. Yes

2. No

8. Don't Know/Not Sure

9. Refused

IF (ANS > 1) SKP Q12_16a

Q: Q12_12 ++++++++++++++++++++++++++++++++++++++++

Does this person currently live with you?

1. Yes

2. No

8. Don't Know/Not Sure

9. Refused

Q: Q12_13 ++++++++++++++++++++++++++++++++++++++++

How is this person related to you?

11. Spouse

12. Daughter

13. Son

14. Daughter‑in‑law

15. Son‑in‑law

16. Sister

17. Brother

18. Granddaughter

19. Grandson

20. Niece

21. Nephew

22. Mother

23. Father

24. Grandmother

25. Grandfather

26. Other relative Specify:

88. Don't Know/Not Sure

99. Refused

Q: Q12_14 ++++++++++++++++++++++++++++++++++++++++

How many hours per week do you spend providing care to this person? This includes any kind of help such as watching your family member, dressing or bathing this person, arranging care, or providing transportation?

_ _ _ Enter hours per week

888 Don't Know/Not Sure

999 Refused

Q: Q12_15 ++++++++++++++++++++++++++++++++++++++++

How much of a mental or emotional strain is it on you to provide this care? Would you say no strain, some strain or a lot of strain?

1. No strain

2. Some strain

3. A lot of strain

8. Don't Know/Not Sure

9. Refused

Q: Q12_16a ++++++++++++++++++++++++++++++++++++++++

Is your annual household income from all sources less than $25,000?

1. Yes

2. No

8. Don't Know/Not Sure

9. Refused

IF (ANS = 1) SKP Q12_16b

IF (ANS = 2) SKP Q12_16f

IF (ANS > 7) SKP Q13_1

Q: Q12_16b ++++++++++++++++++++++++++++++++++++++++

Is your annual household income from all sources less than $20,000?

1. Yes

2. No

8. Don't Know/Not Sure

9. Refused

IF (ANS = 1) SKP Q12_16c

IF (ANS = 2) SKP Q13_1

IF (ANS > 7) SKP Q13_1

Q: Q12_16c ++++++++++++++++++++++++++++++++++++++++

Is your annual household income from all sources less than $15,000?

1. Yes

2. No

8. Don't Know/Not Sure

9. Refused

IF (ANS = 1) SKP Q12_16d

IF (ANS = 2) SKP Q13_1

IF (ANS > 7) SKP Q13_1

Q: Q12_16d ++++++++++++++++++++++++++++++++++++++++

Is your annual household income from all sources less than $10,000?

1. Yes

2. No

8. Don't Know/Not Sure

9. Refused

IF (ANS = 1) SKP Q12_16e

IF (ANS = 2) SKP Q13_1

IF (ANS > 7) SKP Q13_1

Q: Q12_16e ++++++++++++++++++++++++++++++++++++++++

Is your annual household income from all sources less than $5,000?

1. Yes

2. No

8. Don't Know/Not Sure

9. Refused

SKP Q13_1

Q: Q12_16f ++++++++++++++++++++++++++++++++++++++++

Is your annual household income from all sources greater than $35,000?

1. Yes

2. No

8. Don't Know/Not Sure

9. Refused

IF (ANS = 2) SKP Q13_1

IF (ANS > 7) SKP Q13_1

Q: Q12_16g ++++++++++++++++++++++++++++++++++++++++

Is your annual household income from all sources greater than $50,000?

1. Yes

2. No

8. Don't Know/Not Sure

9. Refused

IF (ANS = 2) SKP Q13_1

IF (ANS > 7) SKP Q13_1

Q: Q12_16h ++++++++++++++++++++++++++++++++++++++++

Is your annual household income from all sources greater than $75,000?

1. Yes

2. No

8. Don't Know/Not Sure

9. Refused

IF (ANS = 2) SKP Q13_1

IF (ANS > 7) SKP Q13_1

Q: Q12_16i ++++++++++++++++++++++++++++++++++++++++

Is your annual household income from all sources greater than $150,000?

1. Yes

2. No

8. Don't Know/Not Sure

9. Refused

**SECTION 13: Contact Information**

Q: Q13_1 ++++++++++++++++++++++++++++++++++++++++

My last question is about scheduling the in‑home exam. Someone will call you in the next few days to schedule that visit. What is the best time of day for them to call: morning, afternoon or evening?

1. Morning

2. Afternoon

3. Evening

8. Don't Know/Not Sure

9. Refused

IF (ANS = 1) CALLTIME = 1

IF (ANS = 2) CALLTIME = 2

IF (ANS = 3) CALLTIME = 3

CMDO CALLTIME "Calltime" 1

C: Address verification added 11/6/03

Q: Q13_2

Lastly, I just want to verify that we have your correct address.

The address we have for you is:

CATI SHOW STREET ADDRESS FROM SAMPLE FILE

CATI SHOW CITY FROM SAMPLE FILE

CATI SHOW STATE FROM SAMPLE FILE

CATI SHOW ZIP FROM SAMPLE FILE

Is this correct?

1. Yes

2. No

Q: Q13_3a

Please give me the correct address:

Q13_3A Street:

Q13_3B City:

Q13_3C State:

Q13_3D Zip:

Q: CLOSE ++++++++++++++++++++++++++++++++++++++++

Thank you again for participating in this important research project. I appreciate the time you've spent on the phone with me today and someone should contact you soon to set a day and time for your in‑home exam. Have a great day!

INTERVIEWER: Enter your 3 digit id number and hang up the telephone

Q: CLOSE2 ++++++++++++++++++++++++++++++++++++++++

INTERVIEWER: Please rate the respondent on the following three scales:

Hearing?

1. No problem

2. Slight problem

3. Moderate problem

4. Big problem

Q: QCLOSE3 ++++++++++++++++++++++++++++++++++++++++

Cooperation?

1. No problem

2. Slight problem

3. Moderate problem

4. Big problem

Q: QCLOSE4 ++++++++++++++++++++++++++++++++++++++++

Ability to understand the questions?

1. No problem

2. Slight problem

3. Moderate problem

4. Big problem

INTDATE = SYSDATE

CMDO SELADTF "FirstName"

CMDO SELADTL "LastName"

CMDO PHON1 "PhoneNum"

CMDO IDENT "FirmName"

CMDO Sample "Sample" 1

CMDO Race "Race" 1

CMDO Region "Region" 1

DISPOS = 50 CPL
