# Supplementary material for: Assessment tools for unrecognized myocardial infarction: a cross-sectional analysis of the REasons for geographic and racial differences in stroke population
Source: BMC Cardiovasc Disord. 2013 Mar 26;13:23. doi: 10.1186/1471-2261-13-23 (PMC3617994; doi:10.1186/1471-2261-13-23)
Supplement: Additional file 2: Table S1 — Characteristics considered in the expanded assessment tool by unrecognized myocardial infarction status. [file 1471-2261-13-23-S2.docx]

Additional Table 1. Characteristics considered in the expanded assessment tool by unrecognized myocardial infarction status.

|  | UMI  (n = 740) | No MI  (n = 15,913) | p-value |
| --- | --- | --- | --- |
| *Demographics* |  |  |  |
| Age (years) | 66.7 (9.9) | 63.2 (9.6) | <0.001 |
| Female (%) | 61.8 | 65.8 | 0.03 |
| African-American (%) | 42.2 | 41.3 | 0.66 |
| Region (%) |  |  | 0.49 |
| Stroke Belt | 34.3 | 34.6 |  |
| Stroke Buckle | 22.7 | 24.3 |  |
| Nonbelt | 43.0 | 41.0 |  |
| Urbanization (%) |  |  | 0.42 |
| Less than 25% | 18.5 | 20.3 |  |
| 25%-75% | 9.9 | 10.3 |  |
| More than 75% | 71.6 | 69.4 |  |
| Education (%) |  |  | 0.008 |
| Less than high school | 12.9 | 10.3 |  |
| High school | 28.2 | 25.5 |  |
| Some college | 23.4 | 27.9 |  |
| College graduate | 35.6 | 36.3 |  |
| Income (%) |  |  | <0.001 |
| Less than $20,000 | 22.0 | 15.7 |  |
| $20,000-$34,999 | 22.6 | 22.8 |  |
| $35,000-$74,999 | 26.8 | 30.8 |  |
| $75,000 and above | 15.4 | 18.1 |  |
| Refused | 13.2 | 12.6 |  |
| Relationship status (%) |  |  | <0.001 |
| Married | 53.5 | 59.1 |  |
| Single | 6.6 | 5.8 |  |
| Widowed | 24.6 | 17.7 |  |
| Divorced | 12.8 | 15.0 |  |
| Other | 2.4 | 2.4 |  |
| Insurance (%) | 92.8 | 92.3 | 0.62 |
| *Health behaviors* |  |  |  |
| Aspirin use (%) | 39.6 | 36.3 | 0.07 |
| Alcohol use (%) |  |  | 0.40 |
| None | 64.0 | 62.6 |  |
| Moderate | 32.8 | 33.3 |  |
| Heavy | 3.2 | 4.1 |  |
| Smoking status (%) |  |  | 0.003 |
| Never smoker | 45.1 | 50.0 |  |
| Past smoker | 36.8 | 35.9 |  |
| Current smoker | 18.1 | 14.1 |  |
| Exercise (%) |  |  | 0.003 |
| None | 39.5 | 33.4 |  |
| 1-3 times per week | 34.8 | 37.8 |  |
| 4 or more times per week | 25.8 | 28.8 |  |
| TV/video watching (%) |  |  | 0.92 |
| None | 0.9 | 0.9 |  |
| 1-6 hours per week | 13.0 | 13.5 |  |
| 1 hour per day | 8.0 | 7.0 |  |
| 2 hours per day | 21.5 | 22.4 |  |
| 3 hours per day | 26.2 | 27.0 |  |
| ≥4 hours per day | 30.5 | 29.2 |  |
| Ever forget to take medications (%) | 25.5 | 22.5 | 0.06 |
| Ever careless in taking medications (%) | 3.8 | 3.3 | 0.45 |
| Ever miss medications when feeling better (%) | 5.4 | 5.5 | 0.88 |
| Ever miss taking medications when feeling sick (%) | 3.8 | 3.5 | 0.65 |
| *Medical history* |  |  |  |
| History of stroke (%) | 6.8 | 4.3 | 0.002 |
| History of transient ischemic attack (%) | 4.9 | 3.2 | 0.01 |
| History of deep vein thrombosis (%) | 4.6 | 4.6 | 0.93 |
| History of peripheral vascular disease (%) | 1.6 | 1.1 | 0.24 |
| History of dialysis (%) | 0.4 | 0.2 | 0.17 |
| History of falls (%) | 18.7 | 15.5 | 0.02 |
| Self-reported diabetes (%) | 24.6 | 18.9 | <0.001 |
| Unrecognized diabetes (%) | 1.9 | 2.3 | 0.52 |
| Self-reported hypertension (%) | 68.0 | 54.4 | <0.001 |
| Unrecognized hypertension (%) | 4.6 | 5.1 | 0.58 |
| Current use of antihypertensives (%) | 64.0 | 49.5 | <0.001 |
| Self-reported dyslipidemia (%) | 50.1 | 49.3 | 0.64 |
| Unrecognized dyslipidemia (%) | 16.1 | 13.8 | 0.07 |
| Family history of myocardial infarction (%) | 31.1 | 33.7 | 0.13 |
| *Participant reported health scales* |  |  |  |
| Self-reported health (%) |  |  | <0.001 |
| Poor | 4.1 | 2.3 |  |
| Fair | 18.4 | 12.9 |  |
| Good | 32.9 | 34.6 |  |
| Very good | 31.3 | 32.7 |  |
| Excellent | 13.4 | 17.5 |  |
| Cohen’s perceived stress score (points) | 3.5 (3.0) | 3.3 (2.9) | 0.03 |
| CESD-4 (points) | 1.3 (2.2) | 1.1 (2.0) | 0.10 |
| SF-12 physical health (points) | 45.5 (11.0) | 47.4 (10.1) | <0.001 |
| SF-12 mental health (points) | 53.6 (8.7) | 53.9 (8.4) | 0.32 |
| *Participant reported symptoms* |  |  |  |
| Stroke symptoms (%) | 13.9 | 14.1 | 0.87 |
| Wake up because of breathlessness (%) | 12.5 | 8.2 | <0.001 |
| Need more than 1 pillow to sleep (%) | 12.6 | 10.5 | 0.07 |
| *Clinical measurements* |  |  |  |
| Body mass index (kg/m^2^) | 29.0 (6.9) | 29.4 (6.3) | 0.10 |
| HDL cholesterol (mg/dL) | 55.0 (18.4) | 54.4 (16.4) | 0.31 |
| LDL cholesterol (mg/dL) | 112 (35) | 115 (35) | 0.01 |
| Triglycerides (mg/dL) | 126 (65) | 123 (62) | 0.13 |
| Systolic blood pressure (mmHg) | 130 (18) | 126 (16) | <0.001 |
| Diastolic blood pressure (mmHg) | 77 (10) | 76 (10) | 0.12 |
| C-reactive protein (mg/L) | 4.6 (7.7) | 4.4 (7.9) | 0.59 |
| Estimated glomerular filtration rate (%) |  |  | <0.001 |
| ≥90 mL/min/1.73 m^2^ | 39.7 | 50.6 |  |
| 60-89 mL/min/1.73 m^2^ | 46.5 | 40.9 |  |
| 45-59 mL/min/1.73 m^2^ | 8.2 | 5.7 |  |
| 30-44 mL/min/1.73 m^2^ | 3.2 | 2.0 |  |
| <30 mL/min/1.73 m^2^ | 2.3 | 0.8 |  |
| Albumin to creatinine ratio (%) |  |  | <0.001 |
| <30 mg/g | 79.4 | 87.7 |  |
| 30-300 mg/g | 14.6 | 10.5 |  |
| >300 mg/g | 6.0 | 1.9 |  |
| Heart rate (beats per minute) | 68.1 (12.5) | 66.9 (11.2) | 0.004 |
| White blood cell count | 6.1 (2.1) | 5.9 (2.9) | 0.05 |
| Impaired cognitive status (%) | 8.9 | 6.3 | 0.005 |

Numbers in table are mean (standard deviation) or percent. UMI: unrecognized myocardial infarction; MI: myocardial infarction
