# Supplementary material for: Assessment tools for unrecognized myocardial infarction: a cross-sectional analysis of the REasons for geographic and racial differences in stroke population
Source: BMC Cardiovasc Disord. 2013 Mar 26;13:23. doi: 10.1186/1471-2261-13-23 (PMC3617994; doi:10.1186/1471-2261-13-23)
Supplement: Additional file 3: Table S2 — Odds ratios and c-statistics for unrecognized myocardial infarction in the expanded assessment tool. [file 1471-2261-13-23-S3.docx]

Additional Table 2. Odds ratios and c-statistics for unrecognized myocardial infarction in the expanded assessment tool

|  | Univariate c-statistic | Odds ratio (95% CI)  Unadjusted |
| --- | --- | --- |
| *Demographics* |  |  |
| Age (10 years) | 0.602 | 1.45 (1.35-1.57) |
| Sex | 0.520 |  |
| Male |  | 1.00 (reference) |
| Female |  | 0.84 (0.72-0.98) |
| Race | 0.504 |  |
| White |  | 1.00 (reference) |
| African-American |  | 1.03 (0.89-1.20) |
| Region | 0.512 |  |
| Nonbelt |  | 1.00 (reference) |
| Stroke belt |  | 0.95 (0.80-1.12) |
| Stroke buckle |  | 0.89 (0.74-1.08) |
| Urbanization | 0.512 |  |
| Less than 25% |  | 0.88 (0.73-1.07) |
| 25%-75% |  | 0.93 (0.72-1.19) |
| More than 75% |  | 1.00 (reference) |
| Education | 0.535 |  |
| Less than high school |  | 1.29 (1.01-1.63) |
| High school |  | 1.13 (0.93-1.36) |
| Some college |  | 0.86 (0.70-1.04) |
| College graduate |  | 1.00 (reference) |
| Income | 0.545 |  |
| Less than $20,000 |  | 1.62 (1.31-2.00) |
| $20,000-$34,999 |  | 1.14 (0.92-1.40) |
| $35,000-$74,999 |  | 1.00 (reference) |
| $75,000 and above |  | 0.98 (0.78-1.24) |
| Refused |  | 1.21 (0.94-1.54) |
| Relationship status | 0.543 |  |
| Married |  | 1.00 (reference) |
| Single |  | 1.27 (0.93-1.72) |
| Widowed |  | 1.53 (1.28-1.84) |
| Divorced |  | 0.95 (0.75-1.19) |
| Other |  | 1.13 (0.70-1.83) |
| Health insurance | 0.502 | 1.08 (0.81-1.43) |
| *Health behaviors* |  |  |
| Aspirin use | 0.517 | 1.15 (0.99-1.34) |
| Alcohol use | 0.509 |  |
| None |  | 1.00 (reference) |
| Moderate |  | 0.97 (0.82-1.13) |
| Heavy |  | 0.75 (0.49-1.15) |
| Smoking status | 0.531 |  |
| Never smoker |  | 1.00 (reference) |
| Past smoker |  | 1.13 (0.96-1.33) |
| Current smoker |  | 1.42 (1.16-1.75) |
| Exercise | 0.532 |  |
| None |  | 1.00 (reference) |
| 1-3 times per week |  | 0.78 (0.66-0.93) |
| 4 or more times per week |  | 0.76 (0.63-0.92) |
| TV/video watching | 0.512 |  |
| None |  | 0.95 (0.38-2.35) |
| 1-6 hours per week |  | 0.92 (0.70-1.22) |
| 1 hour per day |  | 1.09 (0.78-1.52) |
| 2 hours per day |  | 0.92 (0.73-1.16) |
| 3 hours per day |  | 0.93 (0.74-1.16) |
| ≥4 hours per day |  | 1.00 (reference) |
| Ever forget to take medications | 0.515 | 1.18 (1.00-1.40) |
| Ever careless in taking medications | 0.503 | 1.16 (0.79-1/71) |
| Ever miss medications when feeling better | 0.501 | 0.98 (0.70-1.35) |
| Ever miss taking medications when feeling sick | 0.502 | 1.09 (0.74-1.61) |
| *Medical history* |  |  |
| History of stroke | 0.512 | 1.60 (1.19-2.16) |
| History of transient ischemic attack | 0.509 | 1.58 (1.11-2.26) |
| History of deep vein thrombosis | 0.500 | 1.02 (0.72-1.45) |
| History of peripheral vascular disease | 0.502 | 1.43 (0.79-2.57) |
| History of dialysis | 0.501 | 2.24 (0.68-7.36) |
| History of falls | 0.516 | 1.24 (1.03-1.51) |
| Self-reported diabetes | 0.528 | 1.40 (1.18-1.66) |
| Unrecognized diabetes | 0.502 | 0.84 (0.49-1.44) |
| Self-reported hypertension | 0.568 | 1.78 (1.52-2.09) |
| Unrecognized hypertension | 0.502 | 0.91 (0.64-1.29) |
| Current use of antihypertensives | 0.572 | 1.81 (1.55-2.12) |
| Self-reported dyslipidemia | 0.504 | 1.04 (0.89-1.20) |
| Unrecognized dyslipidemia | 0.512 | 1.20 (0.98-1.47) |
| Family history of myocardial infarction | 0.513 | 0.89 (0.76-1.04) |
| *Patient reported health scales* |  |  |
| Self-reported health | 0.548 |  |
| Poor |  | 1.83 (1.24-2.72) |
| Fair |  | 1.51 (1.21-1.87) |
| Good |  | 1.00 (reference) |
| Very good |  | 1.01 (0.84-1.21) |
| Excellent |  | 0.81 (0.64-1.02) |
| Perceived stress scale (1 point) | 0.520 | 1.03 (1.00-1.05) |
| CESD-4 (1 point) | 0.520 | 1.03 (1.00 -1.06) |
| SF-12 physical health (1 point) | 0.549 | 0.98 (0.98-0.99) |
| SF-12 mental health (1 point) | 0.510 | 1.00 (0.99-1.00) |
| *Patient reported symptoms* |  |  |
| Stroke symptoms | 0.501 | 0.98 (0.79-1.22) |
| Wake up because of breathlessness | 0.522 | 1.61 (1.28-2.01) |
| Need more than 1 pillow to sleep | 0.510 | 1.23 (0.98-1.54) |
| *Clinical measurements* |  |  |
| Body mass index (1 kg/m^2^) | 0.524 | 0.99 (0.98-1.00) |
| HDL cholesterol (10 mg/dL) | 0.500 | 1.02 (0.98-1.07) |
| LDL cholesterol (10 mg/dL) | 0.528 | 0.97 (0.95-0.99) |
| Triglycerides (10 mg/dL) | 0.517 | 1.01 (1.00-1.02) |
| Systolic blood pressure (10 mmHg) | 0.564 | 1.15 (1.10-1.19) |
| Diastolic blood pressure (10 mmHg) | 0.510 | 1.06 (0.98-1.15) |
| C-reactive protein (1 mg/L) | 0.501 | 1.00 (0.99-1.01) |
| Estimated glomerular filtration rate | 0.564 |  |
| ≥90 mL/min/1.73 m^2^ |  | 1.00 (reference) |
| 60-89 mL/min/1.73 m^2^ |  | 1.45 (1.24-1.70) |
| 45-59 mL/min/1.73 m^2^ |  | 1.84 (1.39-2.45) |
| 30-44 mL/min/1.73 m^2^ |  | 2.06 (1.34-3.16) |
| <30 mL/min/1.73 m^2^ |  | 3.82 (2.27-6.43) |
| Albumin to creatinine ratio (%) | 0.543 |  |
| <30 mg/g |  | 1.00 (reference) |
| 30-300 mg/g |  | 1.54 (1.24-1.91) |
| >300 mg/g |  | 3.53 (2.53-4.94) |
| Heart rate (beats per minute) | 0.525 | 1.01 (1.00-1.02) |
| White blood cell count (1 unit) | 0.528 | 1.01 (1.00-1.03) |
| Impaired cognitive status | 0.513 | 1.46 (1.12-1.89) |
